# Precision engineering for PRRSV resistance in pigs: Macrophages from genome edited pigs lacking CD163 SRCR5 domain are fully resistant to both PRRSV genotypes while maintaining biological function

**DOI:** 10.1371/journal.ppat.1006206

**Published:** 2017-02-23

**Authors:** Christine Burkard, Simon G. Lillico, Elizabeth Reid, Ben Jackson, Alan J. Mileham, Tahar Ait-Ali, C. Bruce A. Whitelaw, Alan L. Archibald

**Affiliations:** 1 The Roslin Institute and Royal (Dick) School of Veterinary Studies, University of Edinburgh, Easter Bush, Midlothian, United Kingdom; 2 The Pirbright Institute, Ash Road, Pirbright, Woking, United Kingdom; 3 Genus plc, DeForest, Wisconsin, United States of America; University of Georgia, UNITED STATES

## Abstract

Porcine Reproductive and Respiratory Syndrome (PRRS) is a panzootic infectious disease of pigs, causing major economic losses to the world-wide pig industry. PRRS manifests differently in pigs of all ages but primarily causes late-term abortions and stillbirths in sows and respiratory disease in piglets. The causative agent of the disease is the positive-strand RNA PRRS virus (PRRSV). PRRSV has a narrow host cell tropism, limited to cells of the monocyte/macrophage lineage. CD163 has been described as a fusion receptor for PRRSV, whereby the scavenger receptor cysteine-rich domain 5 (SRCR5) region was shown to be an interaction site for the virus *in vitro*. CD163 is expressed at high levels on the surface of macrophages, particularly in the respiratory system. Here we describe the application of CRISPR/Cas9 to pig zygotes, resulting in the generation of pigs with a deletion of Exon 7 of the CD163 gene, encoding SRCR5. Deletion of SRCR5 showed no adverse effects in pigs maintained under standard husbandry conditions with normal growth rates and complete blood counts observed. Pulmonary alveolar macrophages (PAMs) and peripheral blood monocytes (PBMCs) were isolated from the animals and assessed *in vitro*. Both PAMs and macrophages obtained from PBMCs by CSF1 stimulation (PMMs) show the characteristic differentiation and cell surface marker expression of macrophages of the respective origin. Expression and correct folding of the SRCR5 deletion CD163 on the surface of macrophages and biological activity of the protein as hemoglobin-haptoglobin scavenger was confirmed. Challenge of both PAMs and PMMs with PRRSV genotype 1, subtypes 1, 2, and 3 and PMMs with PRRSV genotype 2 showed complete resistance to viral infections assessed by replication. Confocal microscopy revealed the absence of replication structures in the SRCR5 CD163 deletion macrophages, indicating an inhibition of infection prior to gene expression, i.e. at entry/fusion or unpacking stages.

## Author summary

Porcine Reproductive and Respiratory Syndrome is an endemic infectious disease of pigs, manifesting differently in pigs of different ages but primarily causing late-term abortions and stillbirths in sows and respiratory disease in piglets. The causative agent of the disease is the positive-strand RNA PRRS virus (PRRSV). PRRSV only infects a specific subset of cells of the innate immune system of the monocyte/macrophage lineage. Previous research found that the virus needs a specific receptor, CD163, in order to make its own membrane fuse with the host cell membrane in an uptake vesicle to release the viral genetic information into the cytosol and achieve a successful infection. CD163 has a pearl-on-a-string structure, whereby the “pearl”/ domain number 5 was found to interact with the virus and allow it to infect a cell. Here we describe how we generated pigs lacking the CD163 subdomain 5 using so-called CRISPR/Cas9 gene editing in zygotes. The pigs were healthy under normal husbandry conditions and other biological functions conducted by the CD163 were found to be intact. We isolated a variety of monocyte and macrophage cells from these pigs and found them to be completely resistant to PRRSV infection.

## Introduction

Porcine reproductive and respiratory syndrome (PRRS) is one of the most economically important infectious diseases affecting pigs worldwide. The “mystery swine disease” was first observed almost simultaneously in North America and in Europe in the late 1980s [1,2]. The causative agent of PRRS was identified to be a virus later named PRRS virus (PRRSV). Infected pigs may present with symptoms involving inappetence, fever, lethargy, and respiratory distress. However, the most devastating effects of PRRSV infection are observed in young piglets and pregnant sows. In pregnant sows an infection with PRRSV can cause a partial displacement of the placenta, leading to full abortions or to death and mummification of fetuses *in utero* [3]. Late-term abortions occur in up to 30% of infected sows with litters containing up to 100% stillborn piglets. Live-born piglets from an antenatal infection are often weak and display severe respiratory symptoms, with up to 80% of them dying on a weekly basis pre-weaning [4,5]. Young piglets infected with PRRSV often display diarrhea and severe respiratory distress caused by lesions in the lung. In pre-weaned piglets the infection may be transmitted via the mammary gland secretions of an infected sow [6]. At this age the infection has a fatal outcome in up to 80% of animals. After weaning mortality rates reduce, but continued economic losses due to reduced daily gain and feed efficiency are often observed [4,7,8]. Due to reduction or loss of pregnancies, death in young piglets, and decreased growth rates in all PRRSV infected pigs it is estimated that more than $650m are lost annually to pork producers in the United States alone [9,10].

PRRSV is an enveloped, plus-strand RNA virus belonging to the *Arteriviridae* family in the order *Nidovirales* [11,12]. The PRRSV genome (~15 kb) encodes at least 12 non-structural and seven structural proteins. The viral RNA is packaged by the nucleocapsid protein N, which is surrounded by the lipoprotein envelope, containing the non-glycosylated membrane proteins M and E, as well as four glycosylated glycoproteins GP2, GP3, GP4, and GP5, whereby GP2, 3, and 4 form a complex [13–17].

PRRSV has a very narrow host range, infecting only specific subsets of porcine macrophages [18–20]. It is unknown yet how widespread PRRSV infections are within the superfamily of the *Suoidea*. Whereby European wild boars have been shown to act as a reservoir for PRRSV [21], little is known about infection in African suids, such as bushpigs and warthogs. *In vitro* virus replication is supported by the African Green Monkey cell line MARC-145. Entry of PRRSV into macrophages has been shown to occur via pH-dependent, receptor mediated endocytosis [22,23]. Various attachment factors and receptors have been indicated to be involved in the PRRSV entry process (reviewed in [24]). Heparan sulphate was identified early as an attachment factor of the virus [25–27]. *In vitro* infection of pulmonary alveolar macrophages (PAMs) but not MARC-145 cells was shown to be inhibited by an antibody targeting CD169 (sialoadhesin), a lectin expressed on the surface of macrophages [28]. Overexpression of CD169 in previously non-permissive PK-15 cells showed internalization but not productive replication of PRRSV [29]. Finally, an *in vivo* challenge of genetically modified pigs in which the CD169 gene had been knocked out revealed no increased resistance to PRRSV infection, suggesting that CD169 is an attachment factor that is not essential for PRRSV infection [30]. Even though cell surface protein expression is a major determinant of PRRSV binding and internalization, there appears to be a redundancy amongst cell surface attachment factors, with the potential for additional, as yet unidentified receptors, being involved [31]. The scavenger receptor CD163, also known as haptoglobin scavenger receptor or p155, is expressed on specific subtypes of macrophages and has been identified as a fusion receptor for PRRSV. The extracellular portion of CD163 forms a pearl-on-a-string structure of nine scavenger receptor cysteine-rich (SRCR) domains and is anchored by a single transmembrane segment and a short cytoplasmic domain [32]. CD163 has a variety of biological functions, including mediating systemic inflammation and the removal of hemoglobin from blood plasma (reviewed in [33,34]). Overexpression of CD163 renders non-susceptible cells permissive to PRRSV infection [35], whereby it was found that CD163 does not mediate internalization but is crucial for fusion [36]. The transmembrane anchoring and an interaction with the SRCR domain 5 (SRCR5) of CD163 were found to be essential for successful infection with PRRSV [34,35]. Recent *in vivo* experiments with CD163 knock-out pigs confirmed that CD163 is required for PRRSV infection [37]. However, as CD163 has important biological functions the complete knockout could have a negative physiological impact on the animal, particularly with respect to inflammation and/or infection by other pathogens. Interestingly, whereas all the other eight SRCR domains have been shown to be involved in different biological functions, no specific role has been associated with SRCR5, other than in PRRSV infection [34]. Therefore, this study aimed to generate pigs with a defined CD163 SRCR5 deletion and to assess the susceptibility of macrophages from these pigs to PRRSV infection.

## Results

### Generation of live CD163 SRCR5 deletion pigs by CRISPR/Cas9 editing in zygotes

**Fig 1:**
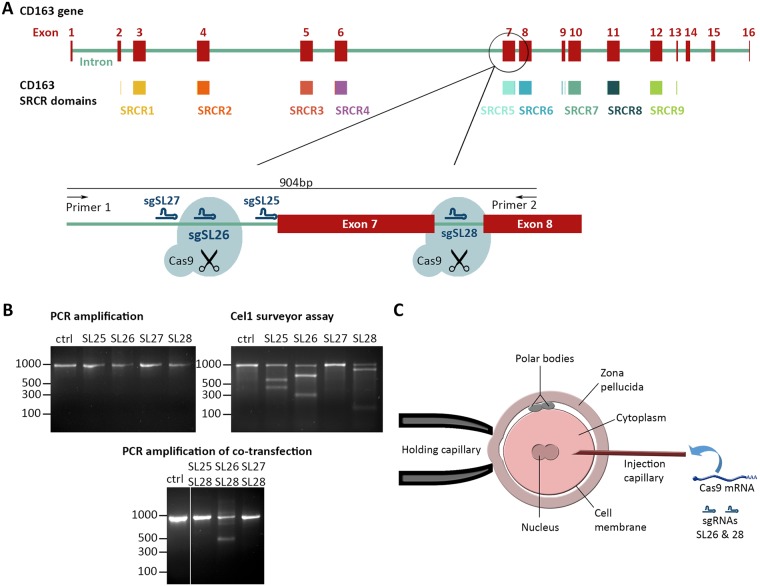
Generation of an exon 7 deletion in CD163 using CRISPR/Cas9. **A)** Schematic of the CD163 gene in the pig genome on chromosome 5. Indicated in red are the 16 exons encoding the CD163 mRNA, in varied colors underneath are the 9 scavenger receptor cysteine-rich (SRCR) domains that form the pearl on a string structure of the CD163 protein. Excision of exon 7 using two guide RNAs (sgSL26 & sgSL28) located in the flanking introns should result in SRCR 5 removal from the encoded protein. Indicated are also the locations of sgRNAs SL25 and SL27. **B)** *In vitro* assessment of guide RNAs sgSL25, sgSL26, sgSL27, and sgSL28. PK15 cells were transfected with either a single plasmid encoding a guide RNA + Cas9 or co-transfected with combination of two such plasmids. Transfected cells were identified by GFP expression and isolated by FACS. Cutting efficiency of single guide RNA transfection was assessed by a Cel1 surveyor assay. Relative efficiency of exon 7 deletion upon double transfection was assessed by PCR. **C)** Schematic of the Cas9/guide RNA injection into zygotes. The injection mix was injected into the cytoplasm of zygotes and contained uncapped, non-polyadenylated guide RNAs sgSL26 and sgSL28, as well as capped, polyadenylated Cas9 mRNA.

The CD163 gene is not correctly represented in the current pig reference genome sequence (Sscrofa10.2) [38]. Through targeted sequencing we established a detailed model of the porcine CD163 locus (S1 File). Briefly, CD163 is encoded by 16 exons with exons 2–13 predicted to encode the SRCR domains of the protein [39]. Interestingly, SRCR5 is predicted to be encoded by one single exon, namely exon 7 (Fig 1A). Thus, an editing strategy was developed to excise exon 7 using the CRISPR/Cas9 genome editing system [40,41]. A combination of two guide RNAs, one located in the intron 5’ to exon 7 and one in the short intron between exons 7 and 8 was predicted to generate a deletion of exon 7, whilst allowing appropriate splicing of the remaining exons. Due to the short length of the intron between exons 7 and 8 (97 bp) only one suitably unique targeting sequence (crRNA) with a corresponding protospacer adjacent motif was identified. Three candidate crRNA sequences were selected in the immediate upstream area of exon 7. All four sequences were assessed *in vitro* for cutting efficiency by transfection of porcine kidney PK15 cells with a plasmid based on px458 [42] encoding the complete single guide sequence (sgRNA), driven by the hU6 promoter, and a CAG promoter driving NLS-Cas9-2A-GFP. Transfected cells were isolated by fluorescence activated cell sorting (FACS) for GFP and cutting efficiency at the target site was assessed using a Cel1 surveyor assay. Three out of four guides were shown to direct cutting of DNA as anticipated (2 upstream and one downstream of exon 7). Following double transfection assay and subsequent PCR analysis it was found that only the combination of guides SL26 and SL28 generated the desired exon 7 deletion in the CD163 gene (Fig 1B). Based on these results the guide combination of sgSL26 and sgSL28 was used for *in vivo* experiments.

sgRNAs SL26 and SL28 were microinjected together with mRNA encoding the Cas9 nuclease into the cytosol of zygotes (Fig 1C). Editing efficiency was assessed in a small number of injected zygotes by *in vitro* culture to the blastocyst stage, genomic DNA extraction, whole genome amplification and PCR amplification across exon 7. The analysis revealed that two out of 17 blastocysts contained a deletion of the intended size and Sanger sequencing confirmed the deletion of exon 7. Edited blastocyst B2 showed a clean deletion and subsequent re-ligation at the cutting sites of sgSL26 and sgSL28, whilst edited blastocyst B14 showed that in addition to the intended deletion there was also a random insertion of 25 nucleotides at the target site (S1 Fig). None of the full length PCR products showed nucleotide mismatches at either cutting site in a T7 endonuclease assay. The overall editing rate in the blastocysts was 11.7%.

To generate live pigs, 24–39 zygotes injected with sgSL26, sgSL28, and Cas9 mRNA were transferred into the oviduct of recipient gilts. A total of 32 live piglets were born and genotyping of ear and tail biopsies revealed that four of the piglets had an exon 7 deletion, corresponding to 12.5% of the total. In addition to the intended deletion of exon 7, three out of the four animals showed insertions of new DNA at the target site probably as a consequence of non-homologous end joining (NHEJ) repair. Pig 347 showed a 2 bp truncation at the sgSL26 cutting site and a 66 bp insertion between the cutting sites, pig 346 showed a deletion of 304 bp after the cutting site of sgSL26, and pig 310 showed a short 9 bp insertion at the cutting sites (S2 Fig). Pig 345 was found to have a precise deletion of exon 7 without insertion or deletion of random nucleotides at the cut sites (S2B & S2D Fig). Interestingly PCR amplification indicated that pigs 310, 345, and 347 were all mosaic for an editing event, with pig 310 having a low frequency heterozygous (one allele edited) compared to unedited cells, whilst pigs 345 and 347 have both homozygous (both alleles edited) and heterozygous cell types (S2A & S2C Fig).

### Genotype and phenotype of F1 generation pigs

**Fig 2:**
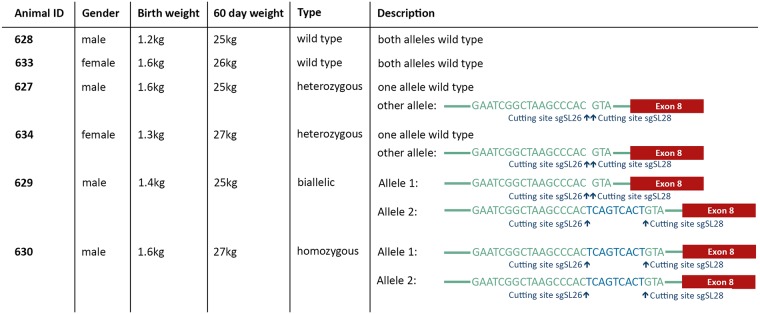
Genotypes and growth of assessed F1 animals.

To generate fully homozygous and heterozygous pigs, 310 was mated with 345. This mating yielded a litter of 6 heterozygous, 2 biallelic/homozygous CD163 SRCR5 deletion (ΔSRCR5), and 4 wild type CD163 piglets (S3 Fig). Sequencing of the animals revealed all the heterozygotes to have inherited their edited allele from 345. Pig 629 was found to be biallelic for the exon 7 deletion with one allele carrying the genotype of 345 and the other allele the one from 310. Interestingly 630 was found to be homozygous for the edited allele with the 9 bp insert between the cutting sites of sgSL26 and sgSL28 as found in the 310 founder / parent (Fig 2). We conclude that this homozygous state has arisen from a gene conversion event in the zygote.

**Fig 3:**
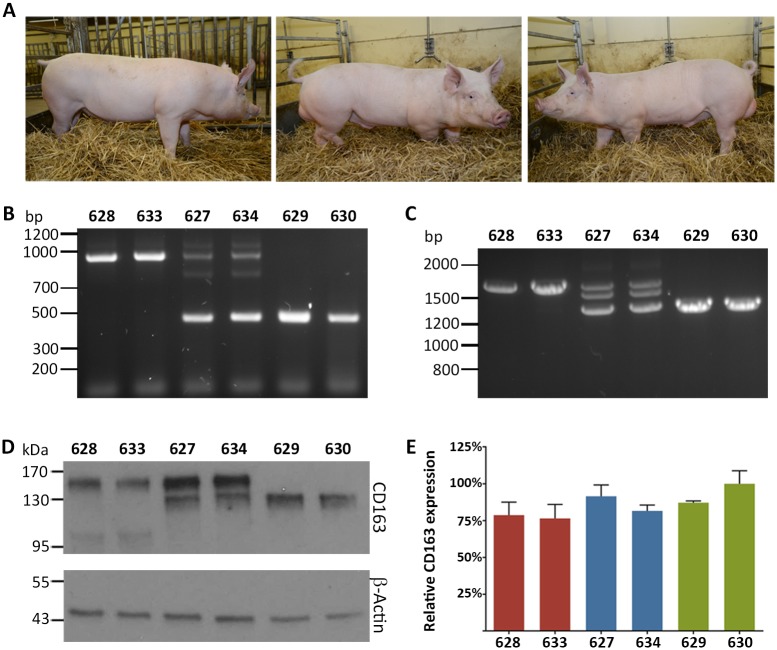
Excision of exon 7 results in an SRCR5 CD163 deletion in pigs. **A)** Representative photos of the male sibling pigs with three different ΔSRCR5 genotypes at 5 months of age. Left, wild type pig 628, middle, heterozygous pig 627, and right, biallelic pig 629. **B)** Genotyping of pulmonary alveolar macrophages (PAMs). DNA was extracted from PAMs and genotype assessed by PCR across Intron 6 to Exon 8. The unmodified genome PCR is predicted to result in a 900 bp product, whilst exon 7 deletion should result in a 450 bp PCR product. **C)** RNA phenotype of pulmonary alveolar macrophages. RNA was extracted from PAMs, converted into cDNA using oligo(dT) primer, and analyzed by PCR across Exons 4–9. The unmodified cDNA should result in a 1686 bp product, whilst the exon 7 deletion is expected to yield a 1371 bp product. **D)** Protein phenotype of CD163 from PAMs. PAM cells were lysed with reducing SDS sample buffer and CD163 expression analyzed by western blot. **E)** CD163 mRNA levels in PAMs. RNA was extracted from the same number of PAM cells, DNA removed by DNase treatment, and RNA quantified by 1-step RT-qPCR. Expression levels were normalized using β-Actin expression levels and to the highest CD163-expressing animal. Error bars represent SEM, n = 3*2.

Animals 627, 628, 629, 630, 633, and 634 were selected for further analysis, representing the various genotypes (wild type, heterozygous, and biallelic/homozygous) and genders. Growth rates of both ΔSRCR5 and heterozygous animals were comparable to wild type animals (Fig 2). Blood samples were taken from all six animals at 10 weeks of age and analyzed by a full blood count conducted by the diagnostics laboratory at the Royal (Dick) School of Veterinary Sciences, University of Edinburgh. The blood counts of all animals were within reference values (S1 table). Size, stature and other morphological features of ΔSRCR5 and heterozygous pigs were comparable to their wild type siblings (Fig 3A).

At 8 weeks of age, pulmonary alveolar macrophages (PAMs) were isolated from all six animals by bronchoalveolar lavage (BAL). DNA was extracted from the PAMs and analyzed by PCR and Sanger sequencing. The PAM genotype confirmed the results obtained from the ear biopsies; 628 and 633 were wild type, 627 and 633 heterozygous, and 629 and 630 ΔSRCR5, respectively. Sequencing of PCR products confirmed that all editing events had resulted in complete deletion of exon 7. Whilst pigs 627 and 633 had a clean deletion of exon 7 with precise religation at the sgSL26 and sgSL28 cutting sites in one allele, 629 had one allele with a clean deletion and one allele with a 9 bp insertion between the sites, and pig 630 had both alleles with the 9 bp insertion (Fig 3B). RNA was extracted from the PAMs, converted into cDNA using oligo(dT) primed reverse transcription, amplified by PCR and analyzed by Sanger sequencing. PCR products spanning exons 4 to 9 showed the expected 315 bp deletion in both heterozygous and ΔSRCR5 animals (Fig 3C). A third fragment situated between the full length and exon 7 deletion band in 627 and 634 was confirmed to be a hybrid of the full length and the exon 7 deletion fragment. This shows that deletion of exon 7 has not disrupted the use of the correct splice acceptor site of exon 8. Expression of CD163 protein was assessed by western blot of PAM lysate. The wild type pigs 628 and 633 expressed the full length protein with a predicted size of 120 kDa but is described to run at roughly 150 kDa [43], likely due to glycosylation, whereby a protein band at roughly 100 kDa may indicate the expression of another isoform, which could correspond to the described human isoform CRA_a or CRA_b (GenBank references EAW88664.1 and EAW88666.1). Heterozygous animals 627 and 634 express both the full-length and the ΔSRCR5 protein (Fig 3D). The band of the full-length protein is clearly stronger, indicating either higher expression of the full-length gene or increased binding of the full-length protein by the polyclonal CD163 antibody used in this study. To further examine this, gene expression was quantified by RT-qPCR on RNA extracted from PAMs and normalized to β-actin expression, demonstrating no significant difference in total CD163 mRNA expression between wild type, heterozygous and ΔSRCR5 animals (Fig 3E).

### Macrophages of ΔSRCR5 pigs are fully differentiated and express macrophage-specific surface proteins

**Fig 4:**
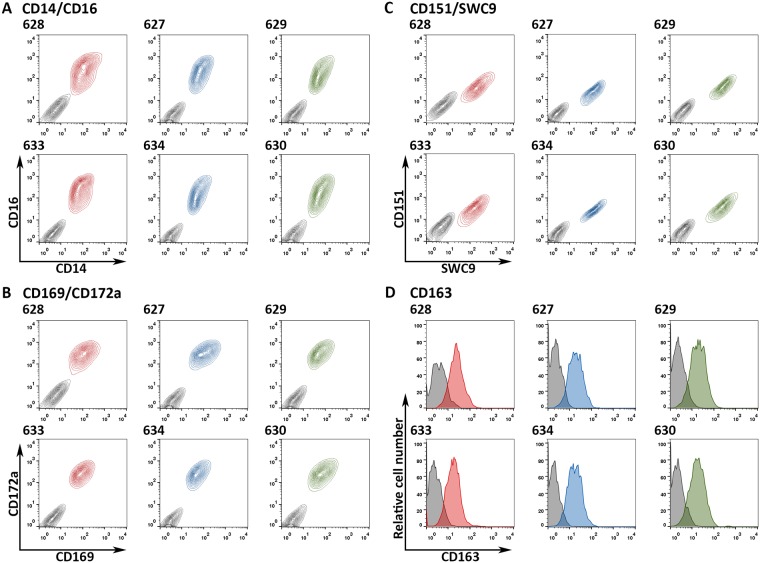
ΔSRCR5 peripheral blood monocyte-derived macrophages (PMMs) are fully differentiated and express macrophage-specific markers. Peripheral blood monocytes were isolated from the blood of the wild type (red), heterozygous (blue), and ΔSRCR5 (green) animals. Following cultivation in the presence of recombinant human CSF1 (rhCSF1) for seven days PMMs were analyzed by FACS. **A)** Co-staining with CD14-FITC and CD16-PE antibodies recognizing the native structure of the proteins (colored contour plots; red wild type, blue heterozygous, green ΔSRCR5) relative to isotype controls (grey). **B)** Co-staining with CD169-FITC and CD172a-PE antibodies recognizing the native structure of the proteins (colored contour plots) relative to isotype controls (grey). **C)** Co-staining with SWC9 (CD203a)-FITC and CD151-RPE antibodies recognizing the native structure of the proteins (colored contour plots) relative to isotype controls (grey). **D)** Staining against the native structure of surface expressed CD163 (colored) relative to an isotype control staining (grey).

To assess the differentiation potential of monocytes into CD163-expressing macrophages we isolated peripheral blood monocytes (PBMCs) from whole blood and then differentiated them into macrophages by CSF1-induction for seven days. Expression of macrophage specific markers was assessed by immunofluorescence labelling and FACS analysis. CD14 and CD16 levels are clear indicators of the differentiation of peripheral blood monocytes with levels of both increasing significantly upon differentiation [44,45]. CD14/CD16 staining of the PMMs from the ΔSRCR5, heterozygous, and wild type animals were all within the previously observed and documented levels [46], with difference being observed between the various genotypes (Fig 4A). CD172a, also known as SIRP α, is expressed at high levels on both monocytes and macrophages [45] and was expressed at high levels in cells from all animals. CD169, described as an attachment factor for PRRSV [29], is not expressed in monocytes but is highly expressed in tissue macrophages [47] and was expressed at expected levels in cells from our animals (Fig 4B). An additional differentiation marker found to be expressed on PMMs is SWC9, also known as CD203a, as well as the putative PRRSV attachment factor CD151 [48,49]. Expression of SWC9 highlighted the full differentiation of the PMMs. CD151 expression together with the previously shown CD169 expression demonstrated that both putative PRRSV attachment factors or receptors are still expressed on macrophages from ΔSRCR5 animals (Fig 4C). As in humans, expression of CD163 in pigs is restricted to monocytes and macrophages. CD163 is expressed at high levels in tissue macrophages, but at low levels in blood monocytes and in bone marrow-derived macrophages [50] (porcine macrophage markers are reviewed in [51]). Both the wild type and the ΔSRCR5 CD163 were recognized on the surface of the PAMs (Fig 4D). This indicates that the ΔSRCR5 version of CD163 is likely to be properly folded as the clone 2A10/11 antibody only recognizes the protein in a non-reduced, native conformation. The medians of CD163 fluorescence intensity of pigs 628, 633, 627, 634, 629, 630 were 23.3, 16.7, 18.3, 16.5, 18.8, and 17.2, respectively, with the isotype control medians ranging from 1.88–3.79. Overall, PBMCs isolated from all animals, independent of their genotype were shown to be fully differentiated into PMMs upon CSF1 induction. They all expressed macrophage-specific surface markers, including CD169, CD151, and CD163, which have putative functions in PRRSV entry.

To confirm the results from the *in vitro* differentiation PAMs were isolated by BAL and characterized for the expression of macrophage-specific surface proteins CD14, CD16, CD169, CD172a, and CD163 as described above. CD14/CD16 staining of the PAMs from the ΔSRCR5, heterozygous, and wild type animals were all within the previously observed and documented levels [46] (S4A Fig). Also CD169 and CD172a were within expected levels, confirming full differentiations (S4B Fig). The medians of CD163 fluorescence intensity of pigs 628, 633, 627, 634, 629, 630 were 35.9, 22.7, 26.4, 24.4, 17.9, and 26.7, respectively, with isotype control medians ranging from 2.13–3.84 (S4C Fig). This indicates slightly higher expression levels of CD163 on PAMs compared to PMMs. Overall, PAMs isolated from all animals, independent of their genotype were shown to be fully differentiated and to express macrophage-specific surface markers, including CD169 and CD163, which have implicated functions in PRRSV entry.

### ΔSRCR5 macrophages are not susceptible to infection with PRRSV genotype 1

PRRSV has two different genotypes with distinct geographic distribution, with genotype 1 being found primarily in Europe and Asia and genotype 2 in the Americas and Asia. The two genotypes show differences in both antigenicity and severity of pathology and show an evolutionary divergence of >15% on a whole genome scale and ∼40% on the nucleotide level between them (reviewed in [52]). Genotype 1 can be further divided into three subtypes, based on the ORF7 sequence and geographical distribution, whereby subtype 1 is pan-European whilst subtypes 2 and 3 are currently limited to Eastern Europe [53]. Here we tested all genotype 1 subtypes of PRRSV, represented by subtype 1 strain H2 (PRRSV H2) [54], subtype 2 strain DAI (PRRSV DAI) [55], and subtype 3 strain SU1-Bel (PRRSV SU1-Bel) [56], originally isolated from the UK, Lithuania, and Belarus, respectively.

**Fig 5:**
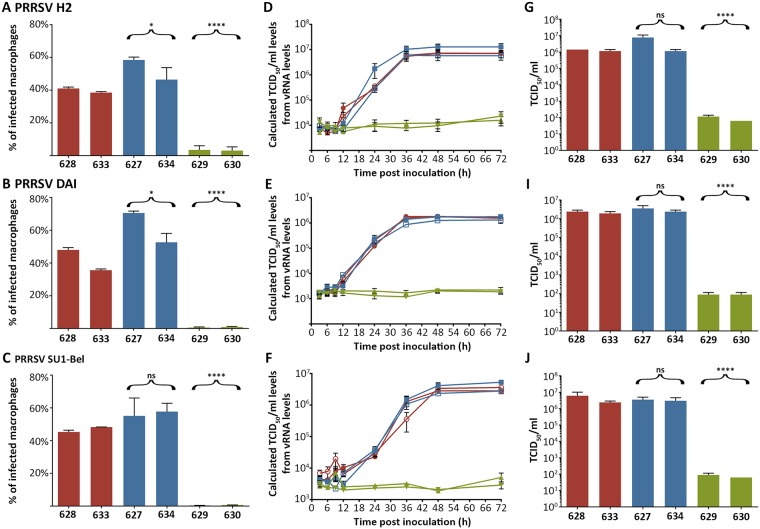
ΔSRCR5 pulmonary alveolar macrophages (PAMs) are not susceptible to infection with PRRSV genotype 1. **A-C)** PAMs from wild type (red), heterozygous (blue), and ΔSRCR5 (green) animals were inoculated at MOI = 1 of PRRSV genotype 1, subtype 1 (strain H2, A), subtype 2 (strain DAI, B), and subtype 3 (strain SU1-Bel, C). 19 hpi cells were detached, fixed and stained with an anti PRRSV-N protein antibody and CD163. Infection was quantified by FACS analysis. Over 98% of infected macrophages were qualified as CD163 positive. Infection levels were statistically analyzed using an unpaired t-test of all wild type against all heterozygous or all biallelic / homozygous data points. Error bars represent SEM, n = 3. **D-F)** Replication growth curves of PRRSV genotype 1, subtype 1 (strain H2, C), subtype 2 (strain DAI, D), and subtype 3 (strain SU1-Bel, F). PAMs from wild type (red, 628 filled circle, 633 open circle), heterozygous (blue, 627 filled square, 633 open square), and ΔSRCR5 (green, 629 triangle pointing down, 630 triangle pointing up) animals were inoculated at MOI = 0.1 of the respective strain. Cell supernatant was collected at indicated time points to measure the released viral RNA by RT-qPCR. Error bars represent SEM, n = 3*2. **G-J)** Quantification of infectious particles produced at 48 hpi by TCID_50_ analysis. Cell supernatant collected at the 48 hpi time point of infection of the time-course experiment was analyzed for infectious viral particle production quantified by TCID_50_. Infection levels were statistically analyzed using an unpaired t-test of all wt against all het or all ΔSRCR5. Error bars represent SEM, n = 3.

PAMs were infected at an MOI = 1 in a single-round infection. 19 hours post inoculation (hpi) the cells were harvested and stained with a FITC-labelled antibody against PRRSV-N protein. Infection levels were assessed by FACS analysis. All three virus subtypes resulted in infection levels of 40–60% in wild type and heterozygous animals, with more than 98% of infected cells being classified as CD163 positive. A slightly higher, statistically significant infection was observed in heterozygous animals infected with PRRSV H2 and DAI. The reason for this is unclear, but may reflect either altered CD163 protein expression profile in heterozygous animals or other, as yet unidentified, genetic properties. By contrast, cells from both ΔSRCR5 animals (629 and 630) were found to be highly resistant to infection in this assay (Fig 5A–5C). A second assay was performed to assess whether virus could replicate in PAMs then infect neighboring cells in a multiple-round infection time course. Cells were inoculated at MOI = 0.1 and supernatant samples collected at indicated time points. Viral RNA was extracted from the supernatants and analyzed by RT-qPCR. For PRRSV H2 and SU1-Bel specific probes and primers against ORF7 were employed. To quantify PRRSV DAI vRNA specific primers against ORF5 and BRYT green dye binding were used due to the limited genome information available on this strain. All wild type and heterozygous animals replicated the virus to similar levels. Virus levels started to rise by 12 hpi and increased exponentially up to 36 hpi when they plateaued. PRRSV SU1-Bel levels reached their plateau at 48 hpi. The quantification limit of the RT-qPCR corresponded to a CT value of 35, which corresponded to 1E4 TCID_50_/ml for PRRSV H2, 1E3 TCID_50_/ml for PRRSV DAI, and 5E3 for PRRSV SU1-Bel. vRNA levels in supernatants from ΔSRCR5 PAMs in this multiple round infection did not increase above the quantification limit (Fig 5D–5F). In order to assess whether infectious virions were produced a TCID_50_ assay was conducted on supernatant collected at 48 hpi, when all three subtypes had reached a plateau. Serial dilutions were started at a 1:10 dilution, corresponding to a detection limit of 63 TCID_50_/ml. Virus produced from PAMs of wild type or heterozygous origin was infectious and levels measured were comparable to those calculated for the vRNA extractions. By contrast, homozygous ΔSRCR5 PAMs did not support virus production at the detection limit of this assay (Fig 5G–5J). In summary, PAMs from ΔSRCR5 animals could not be infected by PRRSV genotype I at a high MOI nor did they replicate the virus over a 72 h time course.

To explore the possibility that PMMs could be a suitable alternative to monitor PRRSV infection and investigate whether ΔSRCR5 PMMs, like PAMs, are resistant to PRRSV infection we tested infectivity with all three genotype 1 subtypes of PRRSV, represented by the strains described above. PMMs were infected and assessed as described for PAMs above in both single-round infections (S5A–S5C Fig) and multiple-round infections (S5D–S5F Fig). The results obtained from PMMs confirmed the ones obtained in PAMs as no replication of PRRSV was observed in cells from ΔSRCR5 animals. Interestingly, PMMs replicated all viruses to higher levels than PAMs, suggesting that PMMs are not only suitable but may in fact be a superior model for *in vitro* infection studies with PRRSV.

As there could be a genetic variation of CD163 within the *Suidae* superfamily we performed an *in vitro* control experiment to assess the susceptibility of warthog (*Phacocherus africanus*) PMMs to PRRSV infection. Interestingly, warthog PMMs were found to be as susceptible to infection with all PRRSV genotype 1 subtypes as the pig PMMs. They all replicated the virus at a similar rate and to comparable titers (S6 Fig). This also shows that the virus poses a threat to African pig breeding countries.

### Peripheral blood monocyte-derived ΔSRCR5 macrophages are not susceptible to infection with PRRSV genotype 2

To assess the infectability of the ΔSRCR5 macrophages with the Asian/American genotype 2 of the virus we selected two strains associated with high virulence, pathology, morbidity and mortality; strain VR-2385, isolated in Iowa in 1992 [57] and MN184, isolated in Minnesota in 2001 [58], (both kindly provided by Prof. Tanja Opriessnig). PMMs from the different CD163 genotypes were subjected to a multiple-round infection. Therefore, cells were inoculated at MOI = 0.1 and supernatant samples collected throughout the progression of infection at 6, 24, 32, 48, and 72 hpi. Viral RNA was extracted from the supernatants and analyzed by RT-qPCR.

**Fig 6:**
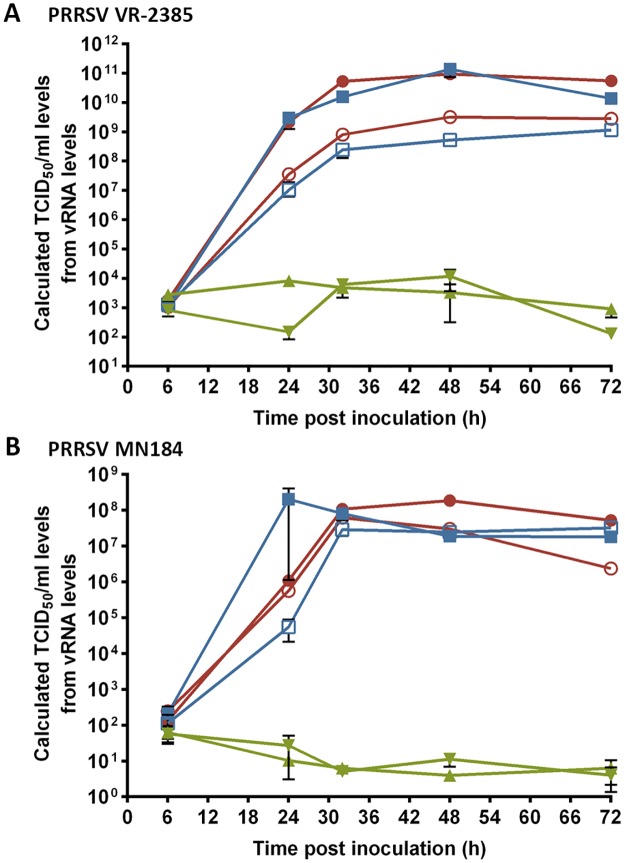
ΔSRCR5 peripheral blood monocyte-derived macrophages (PMMs) are not susceptible to infection with PRRSV genotype 2. Replication of PRRSV on PMMs in long-term infections with genotype 2, **A)** highly virulent strain VR-2385, and **B**) highly pathogenic strain MN-184. PMMs from wild type (red, 628 filled circle, 633 open circle), heterozygous (blue, 627 filled square, 633 open square), and ΔSRCR5 (green, 629 triangle pointing down, 630 triangle pointing up) animals were inoculated at MOI = 0.1 of the respective strain. Cell supernatant was collected at indicated time points to measure the released viral RNA by RT-qPCR. Error bars represent SEM, n = 3*2.

Virus levels for both strains started to rise after the 6 hpi time point and increased exponentially up to 32 hpi when they plateaued (Fig 6A & 6B). The virus amplification in the male homozygous and heterozygous macrophages appears to reach higher levels for strain VR-2385, whereby no difference could be observed for strain MN184. The quantification limit of the RT-qPCR for VR-2385 was found to be at a CT value of 32, which corresponded to 3E2 TCID_50_/ml, for MN184 the quantification limit was at CT value of 36, corresponding to 25 TCID_50_/ml. vRNA levels in supernatants from ΔSRCR5 PMMs in this multiple round infection did not increase above the quantification limit. No replication of PRRSV was observed in ΔSRCR5 animals.

### The arrest in infection of ΔSRCR5 pulmonary alveolar macrophages (PAMs) occurs prior to the formation of the replication/transcription complex

**Fig 7:**
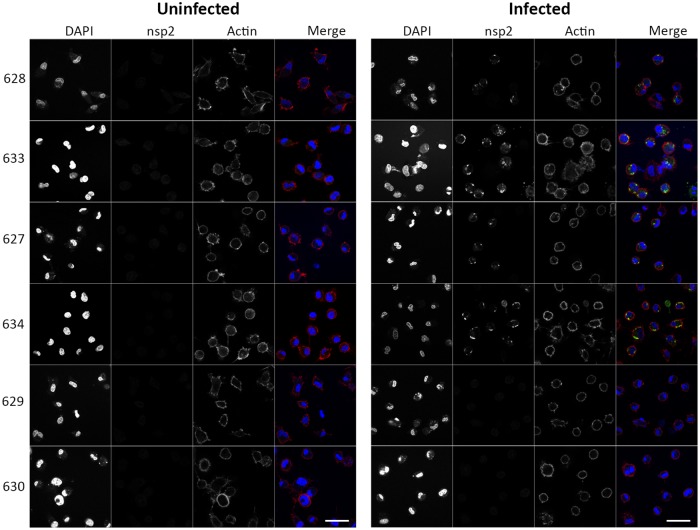
PRRSV infection of ΔSRCR5 pulmonary alveolar macrophages (PAMs) is halted prior to the formation of the replication/transcription complex. PAMs from wild type (top two panels), heterozygous (middle two panels), and ΔSRCR5 (bottom two panels) animals were inoculated at MOI = 2 with PRRSV genotype 1, subtype 3 (strain SU1-Bel). 22 hpi cells were fixed and stained with an anti PRRSV-nsp2 antibody, DAPI, and phalloidin. Scale bar represents 40 μm.

In the porcine kidney cell line PK-15, lacking CD163 expression, transfected with the PRRSV attachment factor CD169 the virus was found to be internalized but not to undergo uncoating [36]. This indicates that CD163, in a close interplay with attachment/internalization factors, plays a major role in the fusion of PRRSV. To assess whether the infection process in ΔSRCR5 macrophages is arrested prior to replication we inoculated PAM cells with all three PRRSV genotype 1 subtypes, represented by the strains described above, at MOI = 2. The inoculum was removed 3 hpi and infection allowed to continue up to 22 hpi. Cells were fixed and stained for the replication-transcription complexes (RTC) formed by PRRSV upon replication initiation. PRRSV nsp2 protein, involved in the formation of double membrane vesicles (reviewed in [59]) was chosen as a representative marker for the RTC. The cells were permeabilized and stained for the presence of PRRSV nsp2. We found that macrophages from both the wild type and the heterozygous animals infected with PRRSV formed RTCs, independent of the subtype. However, in the macrophages from ΔSRCR5 animals no RTC formation was observed. Representative for all strains the SU1-Bel infection is shown in Fig 7, a figure of all infections may be found in S7 Fig This underlines the involvement of CD163 in the entry and uncoating process of PRRSV infection. It also supports the deletion of SRCR5 as an effective method to abrogate PRRSV infection before the virus or viral proteins are amplified.

### ΔSRCR5 peripheral blood monocyte-derived macrophages still function as CD163-dependent hemoglobin-haptoglobin scavengers

In addition to its contribution to PRRSV susceptibility, CD163 has been described to have a variety of important biological functions. CD163 is an erythroblast binding factor, enhancing the survival, proliferation and differentiation of immature erythroblasts, through association with SRCR domain 2 and CD163-expressing macrophages also clear senescent and malformed erythroblasts. SRCR domain 3 plays a crucial role as a hemoglobin (Hb)-haptoglobin (Hp) scavenger receptor. Free Hb is oxidative and toxic; once complexed with Hp it is cleared through binding to SRCR3 on the surface of macrophages and subsequent endocytosis. This prevents oxidative damage, maintains homeostasis, and aids the recycling of iron. Recently, CD163 was also shown to interact with HMGB1-haptoglobin complexes and regulate the inflammatory response in a heme-oxygenase 1 (HO-1) dependent manner [60]. CD163-expressing macrophages were also found to be involved in the clearance of a cytokine named TNF-like weak inducer of apoptosis (TWEAK), with all SRCRs apart from SRCR5 being involved in this process [61]. Soluble CD163 can be found at a high concentration in blood plasma but its function in this niche is still partially unknown (reviewed in [34,62]). However, a recent publication by Akahori et al. showed the TWEAK interaction of CD163 to be involved in ischaemic injury tissue regeneration [63]. Maintaining these biological functions is likely to be crucial to the production of healthy, genetically edited animals. Interestingly, none of the biological functions assigned to CD163 have yet been linked to SRCR5. In order to confirm whether ΔSRCR5 macrophages were still able to take up Hb-Hp complexes we performed a variety of *in vitro* experiments. Hb-Hp complex uptake in PMMs *in vitro* has been investigated extensively in the past, with PMMs able to take up both Hb and Hb-Hp complexes in a CD163-dependent manner and the inducible form of heme oxygenase, HO-1, being upregulated upon Hb-Hp uptake [64,65].

**Fig 8:**
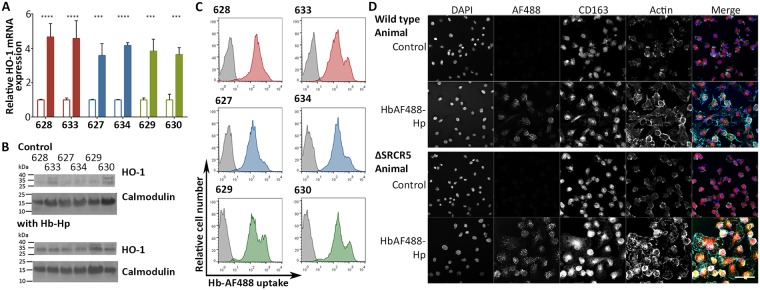
ΔSRCR peripheral blood monocyte-derived macrophages (PMMs) still function as hemoglobin-haptoglobin (Hb-Hp) scavengers. **A)** Induction of HO-1 expression by Hb-Hp uptake. PMMs were incubated for 24 h in presence of 100 μg/ml Hb-Hp. RNA was isolated from cells and levels of heme oxygenase 1 (HO-1) mRNA determined by RT-qPCR (outlined bars uninduced, filled bars Hb-Hp uptake induced; red wild type, blue heterozygous, green ΔSRCR5). Expression levels were normalized using β-Actin expression levels and to the level of unstimulated HO-1 mRNA expression of each animal. Uninduced versus induced levels of HO-1 expression were analyzed by an unpaired t-test. Error bars represent SEM, n = 3. **B**) PMMs were incubated for 24 h in presence of 100 μg/mol Hb-Hp. PMMs were lysed with reducing SDS sample buffer and HO-1 protein expression analyzed by western blot. **C**) Quantification of Hb-Hp uptake. PMMs were incubated in presence of 10 μg/ml Hb_AF488_-Hp for 30 min. Uptake of Hb_AF488_-Hp was measured by FACS analysis (colored contour plots; red wild type, blue heterozygous, green ΔSRCR5) relative to isotype controls (grey). **D)** Visualization of Hb-Hp uptake. PMMs were incubated for 30 min with 10 μg/ml Hb_AF488_-Hp. Cells were fixed, permeabilized and stained against CD163 and with DAPI. Representatively shown are pig 628 as wild type and 630 as ΔSRCR5 animals. Scale bar represents 40 μm.

PBMCs were differentiated into PMMs by CSF1-induction for seven days, following which PMMs were incubated in the presence of Hb-Hp for 24 h to stimulate HO-1 upregulation. The HO-1 mRNA upregulation, assessed by RT-qPCR, increased 2- to 6-fold in the PMMs from all animals (Fig 8A) with no significant difference between the different genotypes. To assess HO-1 levels by western blotting PMMs were incubated in the presence of Hb-Hp for 24 h, lysed using reducing Laemmli sample buffer, and proteins separated by SDS-PAGE. The levels of HO-1 were assessed using a monoclonal antibody against the protein, with a monoclonal antibody against calmodulin as a loading control. HO-1 protein expression was found to be upregulated in all animals, independent of CD163 genotype (Fig 8B). To evaluate the uptake of Hb-Hp directly Hb was labelled with Alexa Fluor 488 (AF488). PMMs were incubated with Hb_AF488_-Hp for 30 min and followed by FACS analysis. Independent of the CD163 genotype, Hb_AF488_-Hp was taken up efficiently by the PMMs with medians of green fluorescence being 329, 305, 329, 366, 340, and 405 for animals 628, 633, 627, 634, 629, and 630, respectively, whilst the background mock-treated cell medians ranged from 2.41–4.74 (Fig 8C). The uptake of Hb_AF488_-Hp into the PMMs was confirmed by confocal microscopy. In a further experiment PMMs were incubated with Hb_AF488_-Hp for 30 min, followed by fixation and staining for CD163. The Hb_AF488_-Hp was found in distinct spots, presumably endosomes, with no obvious co-localization with CD163 (Representative animals wild type 628 and ΔSRCR5 630 shown in Fig 8D, all animals shown in S8 Fig). This lack of co-localization is not surprising as the majority of Hb_AF488_-Hp complexes observed were likely already located in late endosomes and lysosomes. Overall, this data demonstrates that macrophages from ΔSRCR5 animals retain the ability to perform their role as hemoglobin-haptoglobin scavengers.

## Discussion

The results of this study show that live pigs carrying a CD163 SRCR5 deletion are healthy and maintain the main biological functions of the protein, whilst the deletion renders target cells of PRRSV resistant to infection with the virus. By using two sgRNAs flanking exon 7 of *CD163* in CRISPR/Cas9 editing in zygotes we achieved excision of this exon from the genome of pigs yielding a CD163 ΔSRCR5 genotype. The expression of the truncated gene was confirmed by PCR of cDNA, RT-qPCR and western blotting against CD163. Macrophages isolated from the lungs of wild type CD163, heterozygous and ΔSRCR5 animals showed full differentiation and expression of macrophage surface markers characteristic of macrophages isolated from the pulmonary alveolar areas. Assessing infection of PAMs from the different genotype animals in both high dose, single-round infections and low dose, multiple-round infections showed PAMs from ΔSRCR5 pigs to be resistant to infection *in vitro*. The differentiation ability of cells of the monocytes/macrophages lineage from genetically edited CD163 animals was further confirmed by isolation and differentiation of PBMCs. PMMs from ΔSRCR5 pigs were also shown to be resistant to PRRSV infection. PMMs have a crucial biological role, serving as scavengers for Hb-Hp complexes in the blood. Using uptake experiments of fluorescently labelled Hb-Hp complexes as well as gene upregulation assays to monitor the increase of HO-1 upon Hb-Hp stimulation we confirmed that this important biological function is maintained in macrophages isolated from ΔSRCR5 animals.

Using CRISPR/Cas9 editing in zygotes we generated live pigs with exon 7 *CD163* deletions. Editing efficiency was highly variable, dependent on day of the procedure/surgery, in both *in vitro* cultivated blastocysts as well as born animals. However, it needs to be considered that overall numbers are low. The reagents used on the various surgery days were the same and both insemination and surgery times were kept consistent. However, there are many elements in the genome editing process that rely on highly skilled personnel and technical reproducibility. Recent developments in nucleic acid delivery methods for genome editing in zygotes may offer possible solutions to standardize the genome editing process. Various groups recently reported successful genome editing by *in vitro* electroporation of CRISPR/Cas9 regents into zygotes isolated from mice and rats without the necessity to remove the zona pellucida [66–68]. Using electroporation to deliver genome editing reagents *in vivo* Takahasi et al. showed high success with this method in mouse embryos after 1.6 days of gestation [69]. Use of *in vitro* electroporation could standardize the injection process and reduce the requirement for highly trained personnel. As an alternative, *in vivo* electroporation would remove both the requirement for donor animals and the long handling process of zygotes prior to re-implantation, however this procedure has currently only been developed for mice and may prove difficult to adapt to the porcine system (reviewed in [70]). Three out of four of the founder animals were found to be edited in a mosaic pattern, although again caution in over interpretation is to be advised due to the low numbers involved. In animal 310 the mosaicism seems to result from a delayed activity of the CRISPR/Cas9 complex, resulting in an edit of one allele in a single cell at the 4- or 8-cell stage. In animals 345 and 347 an initial editing event appears to occur in one allele at the 1-cell stage and a second editing event, modifying the second allele in one of the cells at the 2-cell stage, resulting in homozygous/heterozygous mosaic animals. Mosaicism has been observed in various studies employing injection of genome editors into porcine zygotes [71–73]. Asymmetric spreading of introduced mRNA seems unlikely following results of Sato et al., who performed *in vitro* EGFP mRNA injections using parthenogenetically activated porcine oocytes, whereby a relatively homogenous fluorescence pattern could be observed [73]. Rather, mosaicism seems to result from Cas9 protein/sgRNA complexes remaining active throughout several cell divisions or delayed mRNA expression possibly triggered by cell division. The former theory is supported by the genotype of 345 and 347, which very likely have developed from an initial editing step in one allele at the one cell stage and editing of the second allele in one of the 2-cell or 4-cell stage cells. To generate more biallelic animals by direct injection of zygotes, a more active reagent set is required. Recent studies indicate that injection of Cas9/sgRNA ribonucleoproteins (RNPs) is more efficient than mRNA injection. Also, RNP injection can be combined with *in vitro* electroporation [74].

The mating of the F0 generation animals 310 and 345 resulted in wild type, heterozygous and biallelic edited animals. This showed that despite mosaicism both animals are germline heterozygous. None of the offspring showed any adverse effect from the genome editing under standard husbandry conditions. Interestingly, the genotype of one of the animals, 630, was consistent with a gene conversion event at the edited CD163 locus. Based on the mechanism of interallelic gene conversion we assume that a homologous recombination occurred in this animal between one allele showing the edited genotype of 345 and the other allele the edited genotype of 310. The gene conversion appears to have occurred at the zygote stage, rendering 630 homozygous for the genotype of 310 (reviewed in [75]).

PRRSV shows a very narrow host cell tropism, only infecting specific porcine macrophage subsets. Isolating these cells from the F1 generation offspring of our genetically edited animals and their wild type siblings we showed that removal of the CD163 SRCR5 domain results in complete resistance of the macrophages towards PRRSV infection. We further demonstrated that macrophages from ΔSRCR5 animals are not only resistant to infection with all European subtypes of genotype 1 but also highly pathogenic and highly virulent strains of the Asian/American genotype 2. This shows that a targeted removal of SRCR5 is sufficient to achieve complete resistance to PRRSV infection *in vitro*. PRRSV attachment factors CD151 and CD169 are still expressed on ΔSRCR5 macrophages underlining that these proteins are not sufficient for PRRSV infection. PRRSV infection on macrophages from the ΔSRCR5 animals was halted before the formation of replication transcription complexes proving CD163 to be involved in the entry or uncoating stage of the PRRSV replication cycle. The ΔSRCR5 macrophages will provide a new tool to study this process in detail in a true-to-life system.

The ΔSRCR5 animals have several advantages over previously described genome edited animals resistant to PRRSV infection. Whitworth et al. generated animals with a premature stop codon in exon 3 of the CD163 gene, resulting in an ablation of CD163 expression [37]. In another recent publication the group replaced the SRCR5 of the porcine CD163 with the human CD163-L1 (hCD163-L1) domain SRCR8, utilizing the strategy employed by van Gorp et al. in their *in vitro* studies of the PRRSV-CD163 interaction [76,77]. However, when replacing the SRCR5 with hCD163-L1 SRCR8 the resulting pigs were found to be susceptible to PRRSV genotype 2 infection. In contrast to both approaches we have demonstrated that specific application of genome editing tools in vivo can be used to efficiently generate PRRSV genotype 1 and genotype 2 resistant animals with precise deletion of exon 7 of CD163, and that these animals retain expression of the remainder of the CD163 protein on the surface of specific differentiated macrophages in a native confirmation. We further showed that the macrophages from these ΔSRCR5 animals retain full differentiation potential, both in PAMs as well as PBMCs stimulated to differentiate by CSF-1 addition, and that macrophages from edited animals retain the ability to perform crucial biological functions associated with CD163 expression, such as hemoglobin-haptoglobin uptake. Furthermore, SRCR5 animals are not transgenic in contrast to hCD163-L1 SRCR8 replacement animals. The hCD163-L1 SRCR8 animals or any variation thereof, which would render the animals resistant to PRRSV genotype 2 as well as genotype 1, bear another risk, which is the adaptation of the virus to the new amino acid sequence of the replacement SRCR domain, thereby turning into a potential human pathogen due to the human sequence used. A removal of SRCR5 has the advantage of removing the virus’s target interaction sequence all together, thereby making it more difficult for the virus to adapt. Overall, this study demonstrates that it is possible to utilize a targeted genome editing approach to render livestock resistant to viral infection, whilst retaining biological function of the targeted gene. Introduction of CD163 SRCR5 deletion animals in pig breeding could significantly reduce the economic losses associated with PRRSV infection.

## Materials and methods

All animal work was approved by The Roslin Institute’s and the University of Edinburgh’s Protocols and Ethics Committees. The experiments were carried out under the authority of U.K. Home Office Project Licenses PPL60/4518 and PPL60/4482 under the regulations of the Animal (Scientific Procedures) Act 1986.

### Cells and viruses

Primary pulmonary alveolar macrophages (PAMs) for the propagation of PRRSV genotype 1, subtype 1 strain H2 (PRRSV H2) [54], subtype 2 strain DAI (PRRSV DAI) [55], and subtype 3 strain SU1-Bel (PRRSV SU1-Bel)[56] were harvested from wild type surplus research animals aged 6–9 weeks as previously described [46]. Briefly, animals were euthanized according to a schedule I method. Lungs were removed and transferred on ice to a sterile environment. PAMs were extracted from lungs by washing the lungs twice with warm PBS, massaging to release macrophages. Cells were collected by centrifugation for 10 min at 400 x g. When necessary red cells were removed using red cell lysis buffer (10 mM KHCO_3_, 155 mM NH_4_Cl, 0.1 mM EDTA, pH 8.0) for 5 min before washing again with PBS. Cells were collected by centrifugation as before and frozen in 90% FBS (HI, GE Healthcare), 10% DMSO (Sigma). Cells were frozen gradually at 1°C/min in a -80°C freezer before being transferred to -150°C. Genotype 2 PRRSV strains VR-2385, isolated in Iowa in 1992 [57] and MN184, isolated in Minnesota in 2001 [58], (both kindly provided by Prof. Tanja Opriessnig [78]) were assessed for infectivity on PAM cells prior to use in infection experiments.

PAMs from the animals 627, 628, 629, 630, 633, and 634 were collected at 8 weeks of age. For this the piglets were sedated using a Ketamine/Azaperone pre-medication mix and anaesthetized with Ketamine/Midazolam. Anesthesia throughout the procedure was maintained using Sevoflurane. PAMs were collected by bronchoalveolar lavage (BAL) through an intubation with an air flow access. Three lung segments were flushed in each animal using 2 x 20 ml PBS. Fluid recovery was between 60–80%. Cells were collected by centrifugation for 10min at 400g from the BAL fluid and frozen as above.

Peripheral blood monocytes (PBMCs) were isolated as described previously [46]. Briefly, blood was collected using EDTA coated vacuum tubes from the jugular vein of the piglets at 10 weeks of age. Blood was centrifuged at 1200 x g for 15 min and buffy coat transferred to PBS. Lymphoprep (Axis-Shield) was overlaid with an equal volume of buffy coat/PBS and centrifuged for 45 min at 400 x g. The mononuclear cell fraction was washed with PBS, cells collected and frozen as described above.

PAM cells were cultivated in RPMI-1640, Glutamax (Invitrogen), 10% FBS (HI, GE Healthcare), 100IU/ml penicillin and 100μg/ml streptomycin (Invitrogen) (cRPMI). PBMCs were cultivated in cRPMI supplemented with rhCSF1 (1×10^4^ units/ml; a gift from Chiron) for 6 days prior to infection.

PK15 cells were cultured in DMEM supplemented with Glutamax (Invitrogen), 10% FBS (HI, GE Healthcare), 100IU/ml penicillin and 100μg/ml streptomycin (Invitrogen).

### Design and *in vitro* cutting efficiency assessment of guide RNAs

Three potential guide RNA sequences were selected in the 200 bp of intron 6 and one in the 97 bp long intron 7. Oligomers (Invitrogen) were ordered, hybridized as previously described [79] then ligated into the BbsI sites of plasmid pSL66 (a derivative of px458 with modifications to the sgRNA scaffold as described by [42]). The generated plasmids contain a hU6 promoter driving expression of the guide RNA sequence and a CBA promoter driving Cas9-2A-GFP with an SV40 nuclear localization signal (NLS) at the N-terminus and a nucleoplasmin NLS at the C-terminus of Cas9. Cutting efficiency of each guide was assessed by transfection of the plasmids into pig PK15 cells using a Neon transfection system (Invitrogen) set at 1400 mV with 2 pulses of 20 mS. 48 hours post-transfection GFP positive cells were collected using a FACS Aria III cell sorter (Becton Dickinson) and cultured for a further 4 days prior to preparation of genomic DNA (DNeasy Blood & Tissues Kit, Qiagen). PCR across the target sites was with oSL46 (ACCTTGATGATTGCGCTCTT) and oSL47 (TGTCCCAGTGAGAGTTGCAG) using AccuPrime Taq DNA polymerase HiFi (Life Technologies) to produce a product of 940 bp. A CelI assay (Transgenomic; Surveyor Mutation Detection Kit) was performed as previously described [80]. Co-transfection of PK15 cells with pairs of plasmids encoding guides flanking exon 7 were carried out as described above with the exception that cells were harvested at 40 hours post-transfection without enrichment for GFP expression. In this instance a truncated PCR product was observed in addition to the 940 bp fragment, indicating deletion of exon 7.

Based on both single and double cutting efficiencies guide RNAs SL26 (GAATCGGCTAAGCCCACTGT), located 121 bp upstream of exon 7, and SL28 (CCCATGCCATGAAGAGGGTA), located 30 bp downstream of exon 7 were selected for *in vivo* experiments.

### Generation of guide RNA and quality assessment

A DNA oligomer fragment containing the entire guide RNA scaffold and a T7 promoter was generated by PCR from the respective plasmid template as follows; a forward primer containing the T7 promoter followed by the first 18 bp of the respective guide RNA and the reverse primers oSL6 (AAAAGCACCGACTCGGTGCC) were used in combination with the Phusion polymerase (NEB). DNA fragments were purified on a 1.5% agarose gel using the MinElute Gel Extraction Kit (Qiagen) according to the manufacturer’s instructions. DNA eluate was further treated with 200 μg/ml Proteinase K (Qiagen) in 10 mM Tris-HCl pH 8.0, 0.5% SDS for 30 min at 50°C followed by phenol/chloroform extraction. Guide RNAs were generated from the resultant DNA fragment using the MEGAshortscript Kit (Thermo Fisher) according to the manufacturer’s instructions. RNA was purified using phenol/chloroform extraction followed by ethanol precipitation and resuspended in EmbryoMax Injection Buffer (Millipore). Purity and concentration of the RNA was assessed using an RNA Screen Tape (Agilent) on an Agilent TapeStation according to the manufacturer’s instructions.

### Zygote injection and transfers

Embryos were produced from Large-White gilts as described previously [80]. Briefly, gilts were superovulated using a regumate/PMSG/Chorulon regime between day 11 and 15 following estrus. Following heat, the donor gilts were inseminated twice in a 6 hour interval. Zygotes were surgically recovered from mated donors into NCSU-23 HEPES base medium, then subjected to a single 2–5 pl cytoplasmic injection with an injection mix containing 50 ng/μl of each guide (SL26 and SL28) and 100 ng/μl Cas9 mRNA (PNA Bio or Tri-Link) in EmbryoMax Injection buffer (Millipore). Recipient females were treated identically to donor gilts but remained unmated. During surgery, the reproductive tract was exposed and 24–39 zygotes were transferred into the oviduct of recipients using a 3.5 French gauge tomcat catheter. Litter sizes ranged from 5–12 piglets.

### *In vitro* assessment genome editing in blastocyst

Uninjected control zygotes and injected surplus zygotes are cultivated in NCSU-23 HEPES base medium, supplemented with cysteine and BSA at 38.5°C for 5–7 days. Blastocysts were harvested at day 7 post cultivation and the genome amplified using the REPLI-g Mini Kit (Qiagen), according to the manufacturer’s instructions. Genotyping was performed as described below.

### Genotyping

Genomic DNA was extracted from ear biopsy or tail clippings taken from piglets at 2 days postpartum using the DNeasy Blood and Tissue Kit (Qiagen). The region spanning intron 6 to exon 8 was amplified using primers oSL46 (ACCTTGATGATTGCGCTCTT) and oSL47 (TGTCCCAGTGAGAGTTGCAG), generating a 904 bp product from the intact allele and a 454 bp product if complete deletion of exon 7 had occurred. PCR products were analyzed by separation on a 1% agarose gel and subsequent Sanger sequencing of all truncated fragments. Fragments corresponding to the wild type length were further analyzed by T7 endonuclease I (NEB) digestion according to the manufacturer’s instructions.

### RNA phenotyping

RNA was isolated from 1E6 PAM cells, isolated by BAL as described above, using the RNeasy Mini Kit (Qiagen), according to the manufacturer’s instructions, including an on-column DNase digestion. First-strand cDNA was synthesized using an Oligo-dT primer in combination with SuperScript II reverse transcriptase (Invitrogen), according to the manufacturer’s instructions. The cDNA was used to assess the RNA phenotype across exons 4 to 9 using primers P0083 (ATGGATCTGATTTAGAGATGAGGC) and P0084 (CTATGCAGGCAACACCATTTTCT), resulting in a PCR product of 1686 bp length for the intact allele and 1371 bp following precise deletion of exon 7. PCR products were analyzed by separation on a 1% agarose gel and subsequent Sanger sequencing of deletion fragments.

### Protein phenotype analysis by western blotting

4E5 PAM cells isolated by BAL were collected by centrifugation at 300 rcf for 10 min. The pellet was resuspended in Laemmli sample buffer containing 100 mM DTT, boiled for 10 min at 95°C and subjected to electrophoresis on 7.5% acrylamide (Bio-Rad) gels. After transfer to a nitrocellulose membrane (Amersham), the presence of cellular proteins was probed with antibodies against CD163 (rabbit pAb, abcam, ab87099) at 1 μg/ml, and β-actin (HRP-tagged, mouse mAb, Sigma, A3854) at 1:2000. For CD163 the blot was subsequently incubated with HRP-labelled rabbit anti-mouse antibody (DAKO, P0260) at 1:5000. Binding of HRP-labelled antibodies was visualized using the Pierce ECL Western Blotting Substrate (Thermo Fisher), according to the manufacturer’s instructions.

### Quantification of CD163 mRNA by RT-qPCR

RNA was isolated from 1E6 PAMs using the RNeasy Mini Kit (Qiagen), according to the manufacturer’s instructions, including an on-column DNase digestion. RNA levels were measured using the GoTaq 1-Step RT-qPCR system (Promega) according to the manufacturers' instructions on a LightCycler 480 (Roche). mRNA levels of CD163 were quantified using primers P0074 (CATGGACACGAGTCTGCTCT) and P0075 (GCTGCCTCCACCTTTAAGTC) and reference mRNA levels of β-actin using primers P0081 (CCCTGGAGAAGAGCTACGAG) and P0082 (AAGGTAGTTTCGTGGATGCC).

### Characterization of macrophages by flow cytometry

PAMs were seeded one day prior to analysis. PBMCs were seeded seven days prior to analysis and differentiated by CSF1 stimulation to yield PBMC-derived macrophages (PMMs). Cells were harvested by scraping with a rubber policeman and fixed in 4% formaldehyde/PBS for 15 min at room temperature. Cells were incubated with blocking solution (PBS, 3% BSA) for 45 min before staining with antibodies. Cells were stained with antibodies targeting either mouse anti pig CD14 (AbD Serotec, MGA1273F, 1:50) and mouse anti pig CD16 (AbD Serotec, MCA2311PE, 1:200), mouse anti pig CD169 (AbD Serotec, MCA2316F, 1:50) and mouse anti pig CD172a (SoutherBiotech, 4525–09, 1:400), mouse anti human CD151 (AbD Serotec, MCA1856PE, 1:50) and mouse anti pig SWC9(CD203a) (AbD Serotec, MCA1973F, 1:50), mouse anti pig CD163 (AbD Serotec, MCA2311PE, 1:50), or mouse IgG1 or an IgG2b negative control (AbD Serotec, MCA928PE,MCA691F, or Sigma, F6397; same concentration as primary Ab). The cells were washed three times with PBS and resuspended in FACS buffer (2% FBS, 0.05M EDTA, 0.2% NaN_3_ in PBS). Gene expression determined by antibody labelling was assessed by FACS analysis on a FACS Calibur (Becton Dickinson) using FlowJo software.

### High MOI single-round infection assay

PAMs were seeded one day prior to infection. PBMCs were seeded seven days prior to infection and differentiated by CSF1 stimulation to yield PBMC-derived macrophages PMMs. Cells were inoculated at MOI = 1 of the respective virus strain (PRRSV H2, DAI, or SU1-Bel) in cRPMI for 3 h at 37°C. The inoculum was replaced by warm cRPMI. At 19 hpi cells were detached by using a cell scraper. Cells were fixed in 4% Formaldehyde (Sigma-Aldrich) in PBS (Gibco) for 15 min at RT, washed with PBS, and subsequently permeabilized in PBS containing 0.1% Triton-X-100 (Alfa Aesar) for 10 min. Cells were incubated with antibody against PRRSV-N (SDOW17-F, RTI, KSL0607, 1:200) and CD163 (AbD Serotec, MCA2311PE, 1:50) or mouse IgG1 negative controls, as described above, in 3% BSA in PBS. The cells were washed three times with PBS and resuspended in FACS buffer. Infection levels, determined by antibody labelling, were assessed by FACS analysis on a FACS Calibur (Benson Dickson) using FlowJo software.

### Low MOI multiple-round infection assay

PAMs were seeded one day prior to infection. PBMCs were seeded seven days prior to infection and differentiated by CSF1 stimulation to yield PMMs. Cells were inoculated at MOI = 0.1 with the respective virus strain (PRRSV H2, DAI, or SU1-Bel) in cRPMI for 3 h at 37°C. Inoculum was removed, cells washed 1x with PBS, and infection continued. At the indicated times post inoculation samples were harvested to be assessed. All samples were frozen and processed once all samples from a time course had been collected.

Viral RNA (vRNA) was extracted from the supernatant samples using the QIAmp Viral RNA Mini Kit according to the manufacturer’s instructions. The viral RNA levels were quantified by RT-qPCR using the GoTaq Probe 1-Step RT-qPCR system (Promega) for PRRSV H2 and SU1-Bel and the GoTaq 1-Step RT-qPCR system (Promega) for PRRSV DAI, VR-2385, and MN184, according to the manufacturer’s instructions. For this the following primers and probes were used: H2 fwd (GATGACRTCCGGCAYC), H2 rev (CAGTTCCTGCGCCTTGAT), H2 probe (6-FAM-TGCAATCGATCCAGACGGCTT-TAMRA), (optimal H2 primer/probe sequences obtained from JP Frossard, AHVLA), SU1-Bel fwd (TCTTTGTTTGCAATCGATCC), SU1-Bel rev (GGCGCACTGTATGACTGACT), SU1-Bel probe (6-FAM-CCGGAACTGCGCTTTCA-TAMRA), DAI fwd (GGATACTATCACGGGCGGTA), DAI rev (GGCACGCCATACAATTCTTA), VR-2385 fwd (CTGGGTAAGATCATCGCTCA), VR-2385 rev (CAGTCGCTAGAGGGAAATGG), MN184 fwd (CTCTCGCGACTGAAGATGAC), MN184 rev (GCCTTGGTTAAAGGCAGTCT). RNA levels were measured on a LightCycler 480 (Roche) using a standard curve generated from vRNA isolates of high titer stocks.

Infectivity of the virus produced was assessed using a TCID_50_ assay of selected time points on PAMs isolated from wild type surplus research animals.

### mRNA and protein levels of heme oxygenase 1 upon Hb-Hp stimulation of PMMs

PBMCs were seeded seven days prior to analysis and differentiated by CSF1 stimulation to yield PMMs. Hemoglobin (Hb, Sigma-Aldrich, A0, H0267) and Haptoglobin (Hp, Sigma Aldrich, Phenotype 2–2, H9762) were mixed in a 1:1 wt/wt ratio in PBS for 15 min on a vertical roller before experimentation. PMMs were incubated with 100 μg/ml Hb-Hp in cRPMI for 24 h at 37°C. Cells were harvested by scraping with a rubber policeman. RNA was isolated from 1E6 cells using the RNeasy Mini Kit (Qiagen), according to the manufacturer’s instructions, including an on-column DNase digestion. RNA levels were measured using the GoTaq 1-Step RT-qPCR system (Promega) according to the manufacturers' instructions on a LightCycler 480 (Roche). mRNA levels of heme oxygenase 1 (HO-1) were quantified using primers P0239 (TACATGGGTGACCTGTCTGG) and P0240 (ACAGCTGCTTGAACTTGGTG) and reference mRNA levels of β-actin using primers P0081 and P0082. For analysis of protein levels of HO-1 cells were collected by centrifugation at 300 rcf for 10 min. The pellet was resuspended in Laemmli sample buffer containing 100 mM DTT, boiled for 10 min at 95°C and subjected to electrophoresis on 12% acrylamide (Bio-Rad) gels. After transfer to a nitrocellulose membrane (Amersham), the presence of cellular proteins was probed with antibodies against HO-1 (mouse mAb, abcam, ab13248, 1:250), and calmodulin (rabbit mAb, abcam, ab45689, 1:1000). The blot was subsequently incubated with HRP-labelled goat anti-rabbit antibody (DAKO, PI-1000) at 1:5000. Binding of HRP-labelled antibodies was visualized using the Pierce ECL Western Blotting Substrate (Thermo Fisher), according to the manufacturer’s instructions.

### Quantification and visualization of hemoglobin-haptoglobin uptake

PBMCs were seeded seven days prior to analysis and differentiated by CSF1 stimulation to yield PMMs. For fluorescence microscopy, cells were seeded on glass cover slips. Hemoglobin (Sigma-Aldrich, A0, H0267) was labeled with Alexa Fluor 488 (AF-488) using a protein labelling kit (Molecular Probes) according to the manufacturer’s instructions. Hb_AF488_ and Hp were mixed in a 1:1 wt/wt ratio in PBS for 15 min on a vertical roller before experimentation. PMMs were incubated with 10 μg/ml Hb_AF488_-Hp in cRPMI for 30 min at 37°C.

For quantification by FACS the cells were collected with a rubber policeman and washed three times with Ca^2+^/Mg^2+^-free PBS to remove surface bound Hb_AF488_-Hp as described previously [65]. Cells were fixed in 4% (wt/v) formaldehyde (Sigma-Aldrich) in PBS (Gibco) for 15 min at RT, washed with PBS, and subsequently permeabilized in PBS containing 0.1% Triton-X-100 (Alfa Aesar) for 10 min. Cells were stained with mouse anti pig CD163 antibody (AbD Serotec, MCA2311PE, 1:50) as described above then washed three times with PBS and resuspended in FACS buffer. Gene expression determined by antibody labelling was assessed by analysis on a FACS Calibur (Becton Dickinson) using FlowJo software.

For immunofluorescence imaging cells were washed three times with Ca^2+^/Mg^2+^-free PBS and fixed in 4% formaldehyde (Sigma-Aldrich) in PBS (Gibco) for 15 min at RT, washed with PBS, then permeabilized in PBS containing 0.1% Triton-X-100 (Alfa Aesar) for 10min. Cells were washed with PBS and incubated with antibody against CD163 (rabbit pAb, abcam, ab87099, 5 μg/ml) in blocking buffer (PBS, 3% FBS) for 1 h, washed, and incubated with secondary goat anti-rabbit AF594 antibody (A11037, 1:100), AF647 phalloidin (A22287, 1:100), and DAPI (1:10,000; all Life Technologies). The samples were analyzed using a confocal laser-scanning microscope (Zeiss LSM-710).

### Immunofluorescence analysis of RTC formation in infected PAMs

PAMs were seeded onto coverslips one day prior to infection. Cells were inoculated at MOI = 2 of the respective virus strain (PRRSV H2, DAI, or SU1-Bel) in cRPMI for 3 h at 37°C. The inoculum was replaced by warm cRPMI. At 19 hpi cells were fixed in 4% formaldehyde (Sigma-Aldrich) in PBS (Gibco) for 15 min at RT, washed with PBS, and permeabilized as described above. Cells were washed with PBS and incubated with antibody against PRRSV nsp2 (A gift from Ying Fang, Kansas State University, [81], 1:400) in blocking buffer for 1 h, washed, and incubated with secondary goat anti-mouse AF488 antibody (A11029, 1:100), AF568 phalloidin (A12380, 1:100), and DAPI (1:10,000; all Life Technologies). The samples were analyzed using a confocal laser-scanning microscope (Zeiss LSM-710).

## Supporting information

S1 Fig Genotypes of blastocyst cultured *in vitro* following zygote injection. **A)** Blastocyst genotype assessed by PCR. The genome from blastocysts was amplified by whole genome amplification and the genotype of the CD163 gene assessed by PCR across intron 6 to exon 8. The unmodified genome PCR is predicted to result in a 900 bp product, whilst exon 7 deletion should result in a 450 bp PCR product. **B)** Specific genotype of blastocysts B2 and B14 assessed by Sanger sequencing of the PCR product.
(TIF)

S2 Fig Genotypes of founder animals. **A)** Genotype of founder animal 310 (f). The genotype of 310 was assessed by PCR across intron 6 to exon 8. DNA template was extracted from two ear biopsies, a tail clipping and from a buffy coat. The unmodified genome PCR is predicted to result in a 900 bp product, whilst the exon 7 deletion should result in a 450 bp PCR product. Displayed as well is the PCR result from one of her unmodified siblings (311) as a control. **B)** Specific genotype of 310 as assessed by Sanger sequencing of the PCR product across intron 6 to exon 8. **C)** Genotype of founder animals 345 (m), 346 (f), and 347 (f). The genotype of the animals was assessed by PCR across intron 6 to exon 8. DNA template was extracted from two ear biopsies, one of them only containing ear tip (epidermis and dermis), buffy coat and pulmonary alveolar macrophages. Genotypes from the different tissue samples reveal a mosaicism of heterozygous and homozygous tissues. Displayed as well are the PCR result from unmodified sibling control animals 342, 343 and 344. **B)** Specific genotype of 345, 346, and 347 as assessed by Sanger sequencing of the PCR product.
(TIF)

S3 Fig Genotypes of litter from 310x345 mating. **A)** The genotype of piglets 627–635 and overlaid/still born piglets was assessed by PCR across intron 6 to exon 8. DNA template was extracted from ear biopsy. The unmodified genome PCR is predicted to result in a 900 bp product, whilst the exon 7 deletion should result in a 450 bp PCR product. **B)** Family tree with indicated genotype. The specific genotypes of 310 and 345 are described in S1 fig On the image the genotype of 310 is represented in red, the one of 345 in blue, grey indicates unmodified (alleles). 310 and 345 are represented as heterozygous despite mosaicisms found in both animals as this represents the genotype found in the germline.
(TIF)

S4 Fig ΔSRCR5 pulmonary alveolar macrophages (PAMs) are fully differentiated and express macrophage-specific markers. PAMs isolated by bronchoalveolar lavage were assessed by staining with various macrophage markers and FACS analysis. **A)** Co-staining with CD14-FITC and CD16-PE antibodies recognizing the native structure of the proteins (colored contour plots; red wild type, blue heterozygous, green ΔSRCR5) relative to isotype controls (grey). **B)** Co-staining with CD169-FITC and CD172a-PE antibodies recognizing the native structure of the proteins (colored contour plots) relative to isotype controls (grey). **C)** Staining against the native structure of surface expressed CD163 (colored) relative to an isotype control staining (grey).
(TIF)

S5 Fig ΔSRCR5 peripheral blood monocyte-derived macrophages (PMMs) are not susceptible to infection with PRRSV genotype 1. **A-C)** PMMs from wild-type (red), heterozygous (blue), and ΔSRCR5 (green) animals were inoculated at MOI = 1 of PRRSV genotype 1, subtype 1 (strain H2, A), subtype 2 (strain DAI, B), and subtype 3 (strain SU1-Bel, C). 19 hpi cells were detached, fixed and stained with anti PRRSV-N protein and CD163 antibodies. Infection was quantified by FACS analysis. Infection levels were statistically analyzed using an unpaired t-test of all wild type against all heterozygous or all ΔSRCR5. Error bars represent SEM, n = 3. **D-F)** Replication of PRRSV on PMMs in long-term infections with genotype 1, subtype 1 (strain H2, **C**), subtype 2 (strain DAI, **D**), and subtype 3 (strain SU1-Bel, **F**). PMMs from wild-type (red, 628 filled circle, 633 open circle), heterozygous (blue, 627 filled square, 633 open square), and ΔSRCR5 (green, 629 triangle pointing down, 630 triangle pointing up) animals were inoculated at MOI = 0.1 of the respective strain. Cell supernatant was collected at indicated time points to measure the released viral RNA by RT-qPCR. Error bars represent SEM, n = 3*2.
(TIF)

S6 Fig Infection of warthog PMMs. Peripheral blood monocytes were isolated from the blood of a warthog. Following cultivation in the presence of rhCSF1 for seven days PMMs were infected or analyzed by FACS. **A-C)** *In vitro* differentiated PMMs from warthog were inoculated at MOI = 0.1 of the respective PRRSV strain. Cell supernatant was collected at indicated time points to measure the released viral RNA by RT-qPCR. Error bars represent SEM, n = 3*2. **D)** Left; Co-staining with CD14-FITC and CD16-PE antibodies recognizing the native structure of the proteins (purple) relative to isotype controls (grey). Middle; Co-staining with CD169-FITC and CD172a-PE antibodies recognizing the native structure of the proteins (purple) relative to isotype controls (grey). Right; Staining against the native structure of surface expressed CD163 (purple) relative to an isotype control staining (grey).
(TIF)

S7 Fig PRRSV infection of ΔSRCR5 pulmonary alveolar macrophages (PAMs) is halted prior to the formation of the replication/transcription complex. PAMs from wild-type (top panels), heterozygous (middle panels), and ΔSRCR5 (bottom panels) animals were inoculated at MOI = 2 with PRRSV genotype 1, subtype 1 (strain H2, top row), subtype 2 (strain DAI, middle row), and subtype 3 (strain SU1-Bel, bottom row). 22 hpi cells were fixed and stained with an anti PRRSV-nsp2 antibody, DAPI, and phalloidin. Scale bar represents 40 μm.
(TIF)

S8 Fig ΔSRCR peripheral blood monocyte-derived macrophages (PMMs) still function as hemoglobin-haptoglobin (Hb-Hp) scavengers. Visualization of Hb-Hp uptake. PMMs were incubated for 30 min with 10 μg/ml Hb_AF488_-Hp. Cells were fixed, permeabilized and stained against CD163 and with DAPI. Scale bar represents 40 μm.
(TIF)

S1 Table Whole blood counts from different genotype animals with reference values. (XLSX)

S1 File CD163 genome locus. (PDF)
