# Supplementary figures and images for: Precision engineering for PRRSV resistance in pigs: Macrophages from genome edited pigs lacking CD163 SRCR5 domain are fully resistant to both PRRSV genotypes while maintaining biological function
Source: PLoS Pathog. 2017 Feb 23;13(2):e1006206. doi: 10.1371/journal.ppat.1006206 (PMC5322883; doi:10.1371/journal.ppat.1006206)

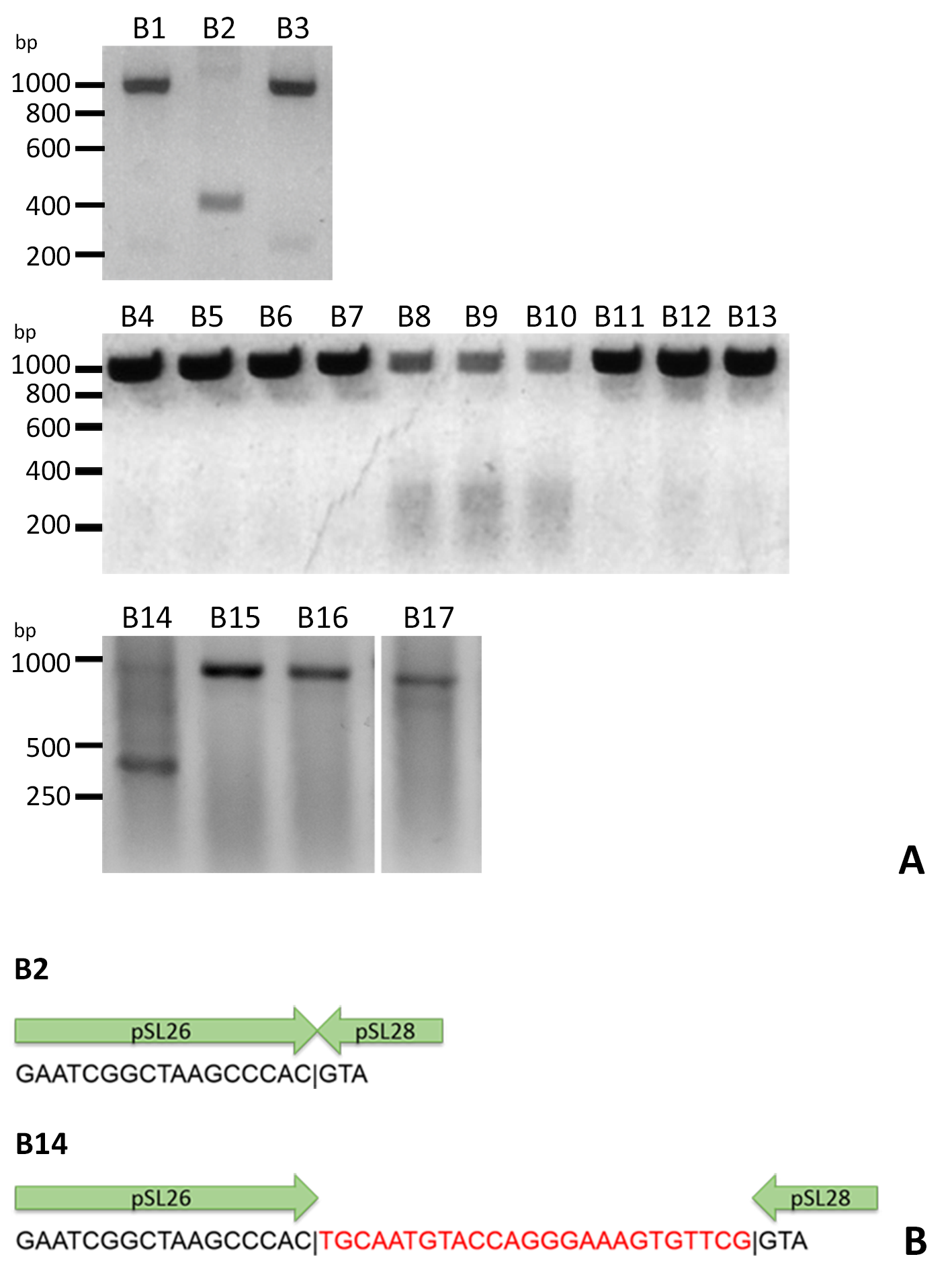

Supplement: S1 Fig — A) Blastocyst genotype assessed by PCR. The genome from blastocysts was amplified by whole genome amplification and the genotype of the CD163 gene assessed by PCR across intron 6 to exon 8. The unmodified genome PCR is predicted to result in a 900 bp product, whilst exon 7 deletion should result in a 450 bp PCR product. B) Specific genotype of blastocysts B2 and B14 assessed by Sanger sequencing of the PCR product. (TIF) [file ppat.1006206.s001.tif]

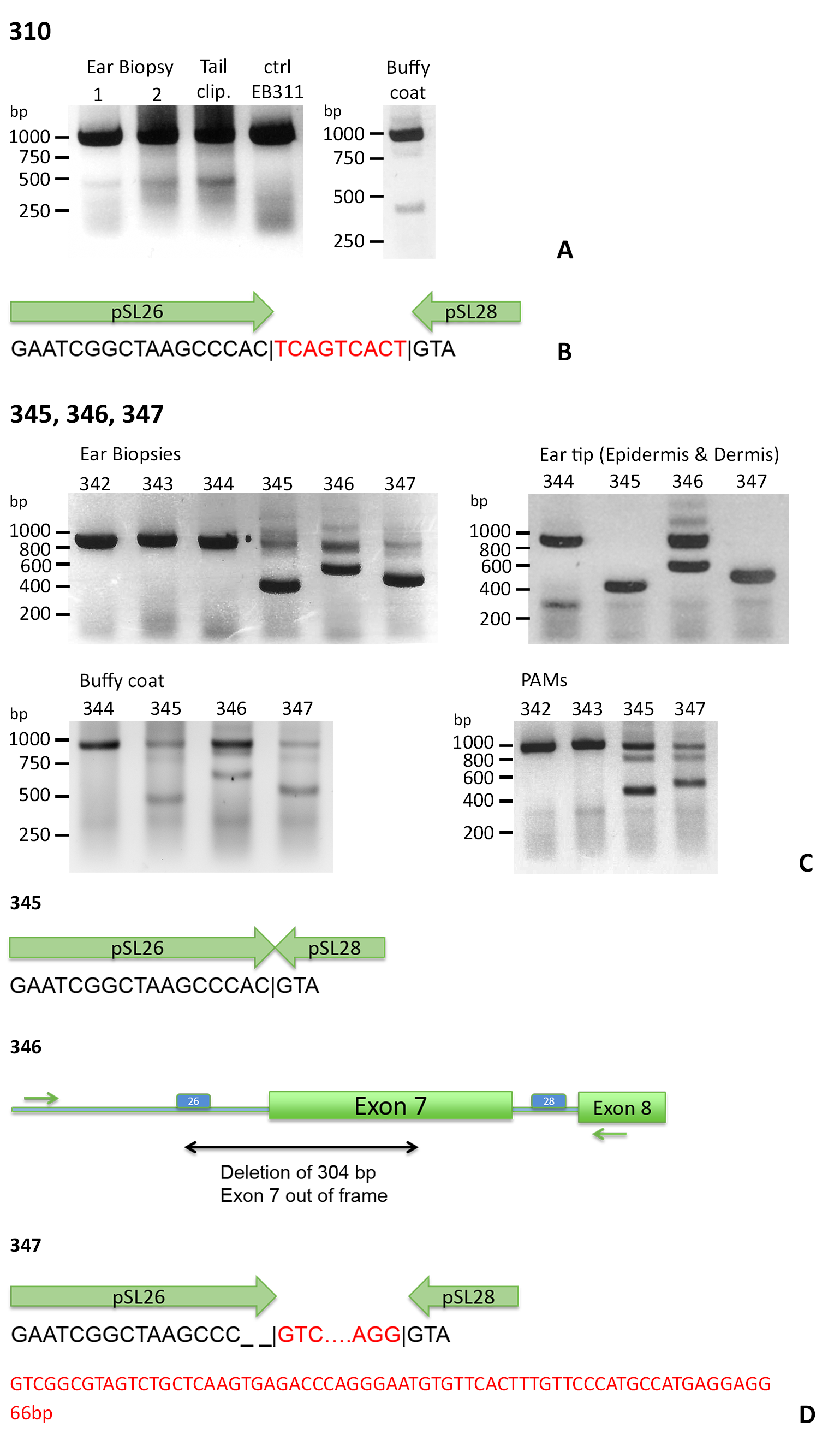

Supplement: S2 Fig — A) Genotype of founder animal 310 (f). The genotype of 310 was assessed by PCR across intron 6 to exon 8. DNA template was extracted from two ear biopsies, a tail clipping and from a buffy coat. The unmodified genome PCR is predicted to result in a 900 bp product, whilst the exon 7 deletion should result in a 450 bp PCR product. Displayed as well is the PCR result from one of her unmodified siblings (311) as a control. B) Specific genotype of 310 as assessed by Sanger sequencing of the PCR product across intron 6 to exon 8. C) Genotype of founder animals 345 (m), 346 (f), and 347 (f). The genotype of the animals was assessed by PCR across intron 6 to exon 8. DNA template was extracted from two ear biopsies, one of them only containing ear tip (epidermis and dermis), buffy coat and pulmonary alveolar macrophages. Genotypes from the different tissue samples reveal a mosaicism of heterozygous and homozygous tissues. Displayed as well are the PCR result from unmodified sibling control animals 342, 343 and 344. B) Specific genotype of 345, 346, and 347 as assessed by Sanger sequencing of the PCR product. (TIF) [file ppat.1006206.s002.tif]

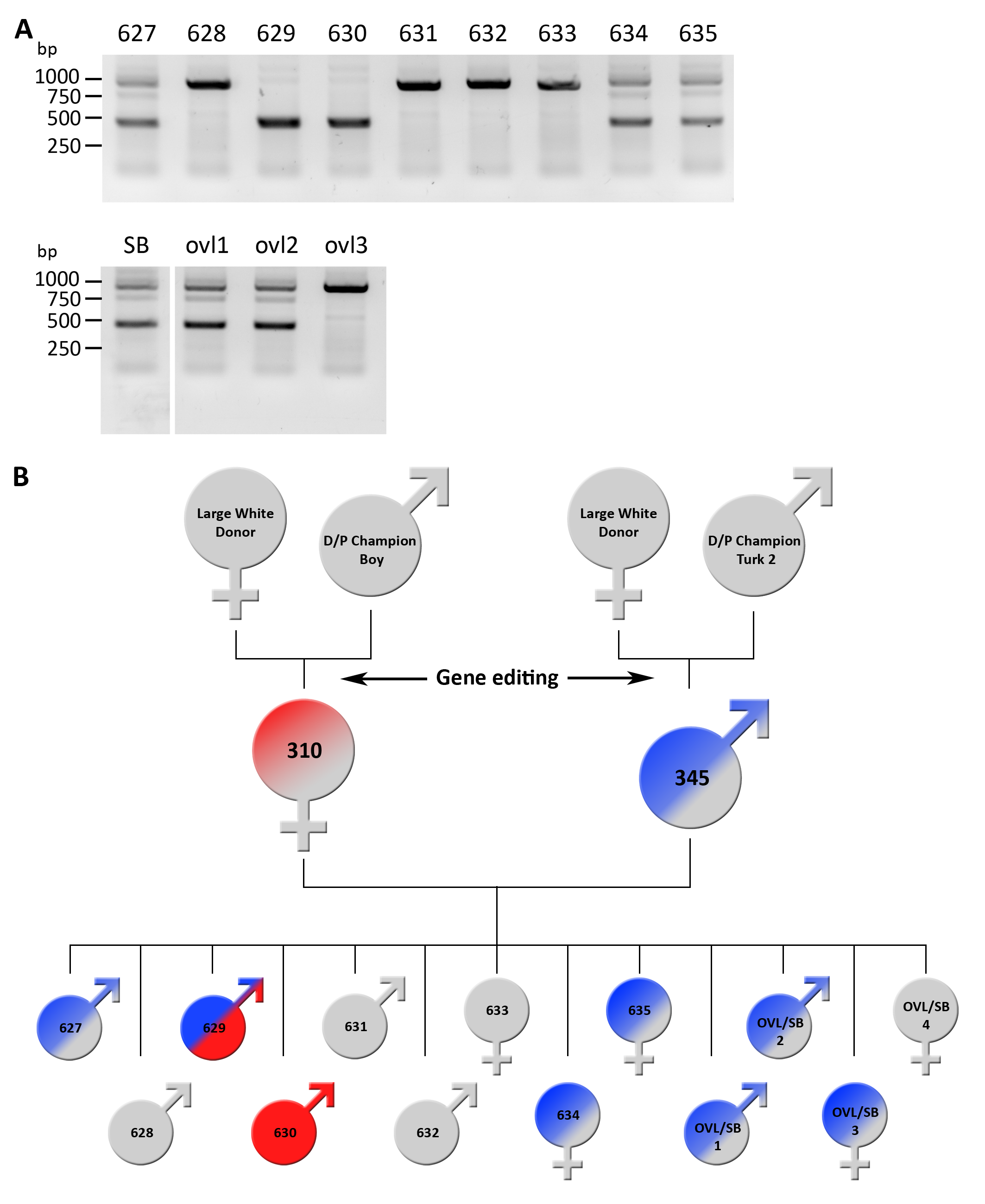

Supplement: S3 Fig — A) The genotype of piglets 627–635 and overlaid/still born piglets was assessed by PCR across intron 6 to exon 8. DNA template was extracted from ear biopsy. The unmodified genome PCR is predicted to result in a 900 bp product, whilst the exon 7 deletion should result in a 450 bp PCR product. B) Family tree with indicated genotype. The specific genotypes of 310 and 345 are described in S1 fig On the image the genotype of 310 is represented in red, the one of 345 in blue, grey indicates unmodified (alleles). 310 and 345 are represented as heterozygous despite mosaicisms found in both animals as this represents the genotype found in the germline. (TIF) [file ppat.1006206.s003.tif]

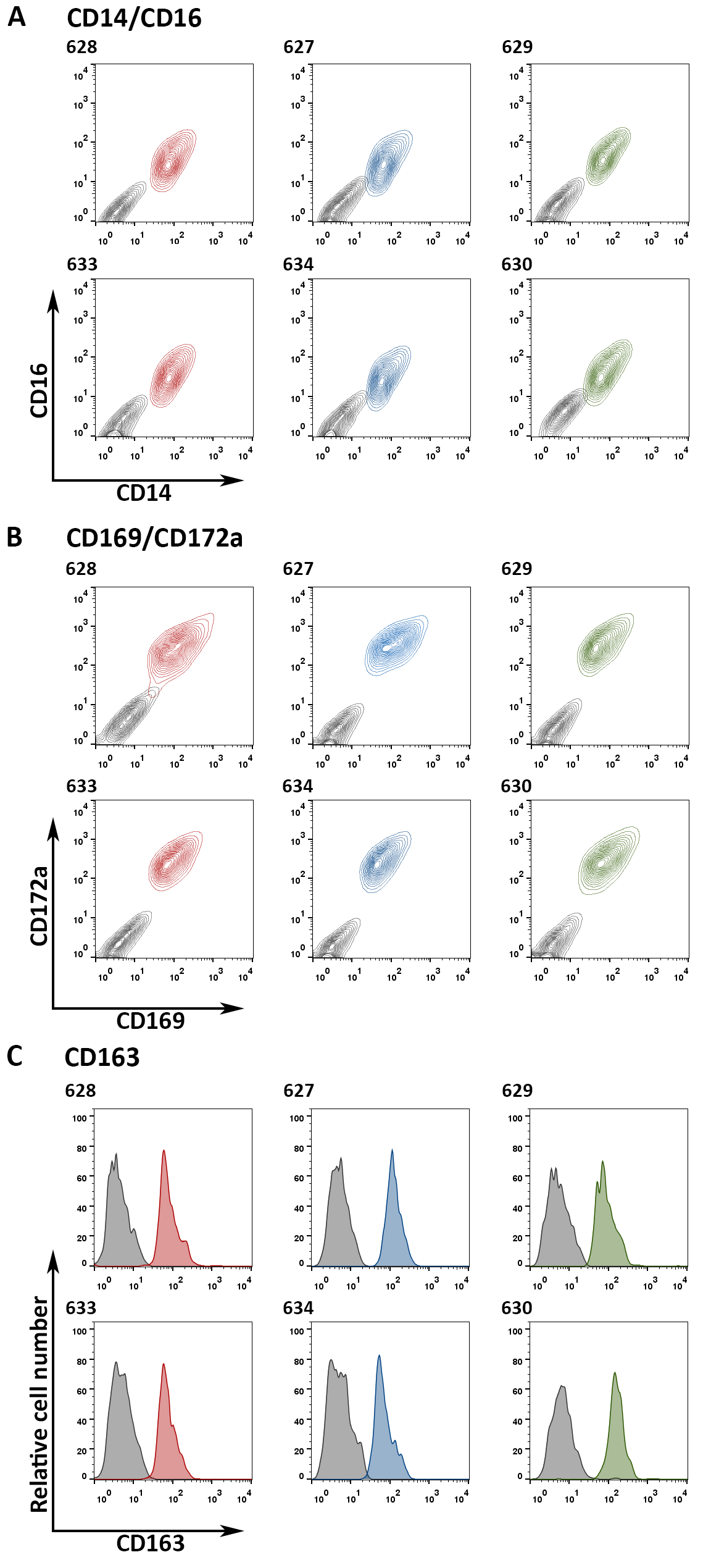

Supplement: S4 Fig — PAMs isolated by bronchoalveolar lavage were assessed by staining with various macrophage markers and FACS analysis. A) Co-staining with CD14-FITC and CD16-PE antibodies recognizing the native structure of the proteins (colored contour plots; red wild type, blue heterozygous, green ΔSRCR5) relative to isotype controls (grey). B) Co-staining with CD169-FITC and CD172a-PE antibodies recognizing the native structure of the proteins (colored contour plots) relative to isotype controls (grey). C) Staining against the native structure of surface expressed CD163 (colored) relative to an isotype control staining (grey). (TIF) [file ppat.1006206.s004.tif]

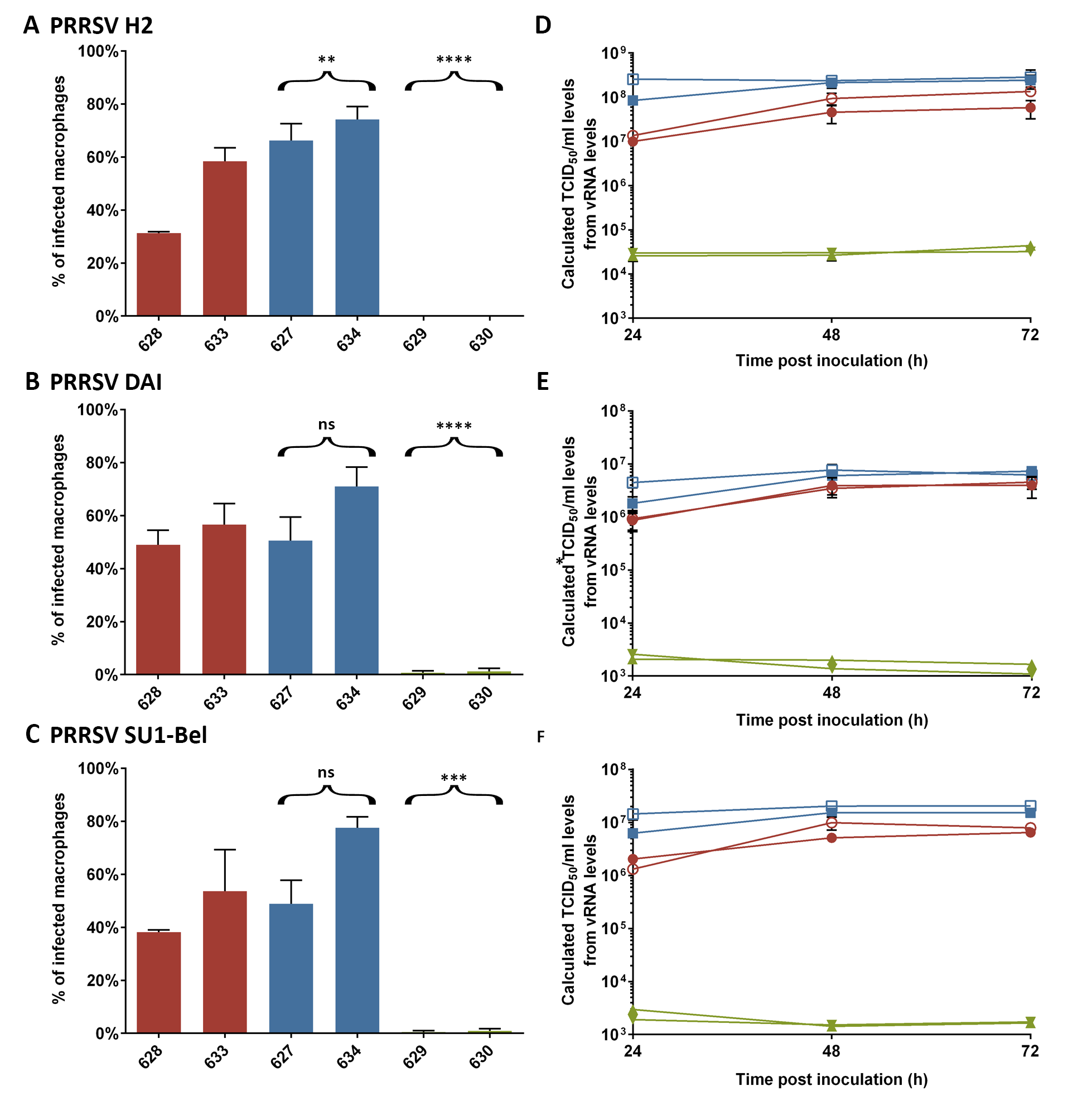

Supplement: S5 Fig — A-C) PMMs from wild-type (red), heterozygous (blue), and ΔSRCR5 (green) animals were inoculated at MOI = 1 of PRRSV genotype 1, subtype 1 (strain H2, A), subtype 2 (strain DAI, B), and subtype 3 (strain SU1-Bel, C). 19 hpi cells were detached, fixed and stained with anti PRRSV-N protein and CD163 antibodies. Infection was quantified by FACS analysis. Infection levels were statistically analyzed using an unpaired t-test of all wild type against all heterozygous or all ΔSRCR5. Error bars represent SEM, n = 3. D-F) Replication of PRRSV on PMMs in long-term infections with genotype 1, subtype 1 (strain H2, C), subtype 2 (strain DAI, D), and subtype 3 (strain SU1-Bel, F). PMMs from wild-type (red, 628 filled circle, 633 open circle), heterozygous (blue, 627 filled square, 633 open square), and ΔSRCR5 (green, 629 triangle pointing down, 630 triangle pointing up) animals were inoculated at MOI = 0.1 of the respective strain. Cell supernatant was collected at indicated time points to measure the released viral RNA by RT-qPCR. Error bars represent SEM, n = 3*2. (TIF) [file ppat.1006206.s005.tif]

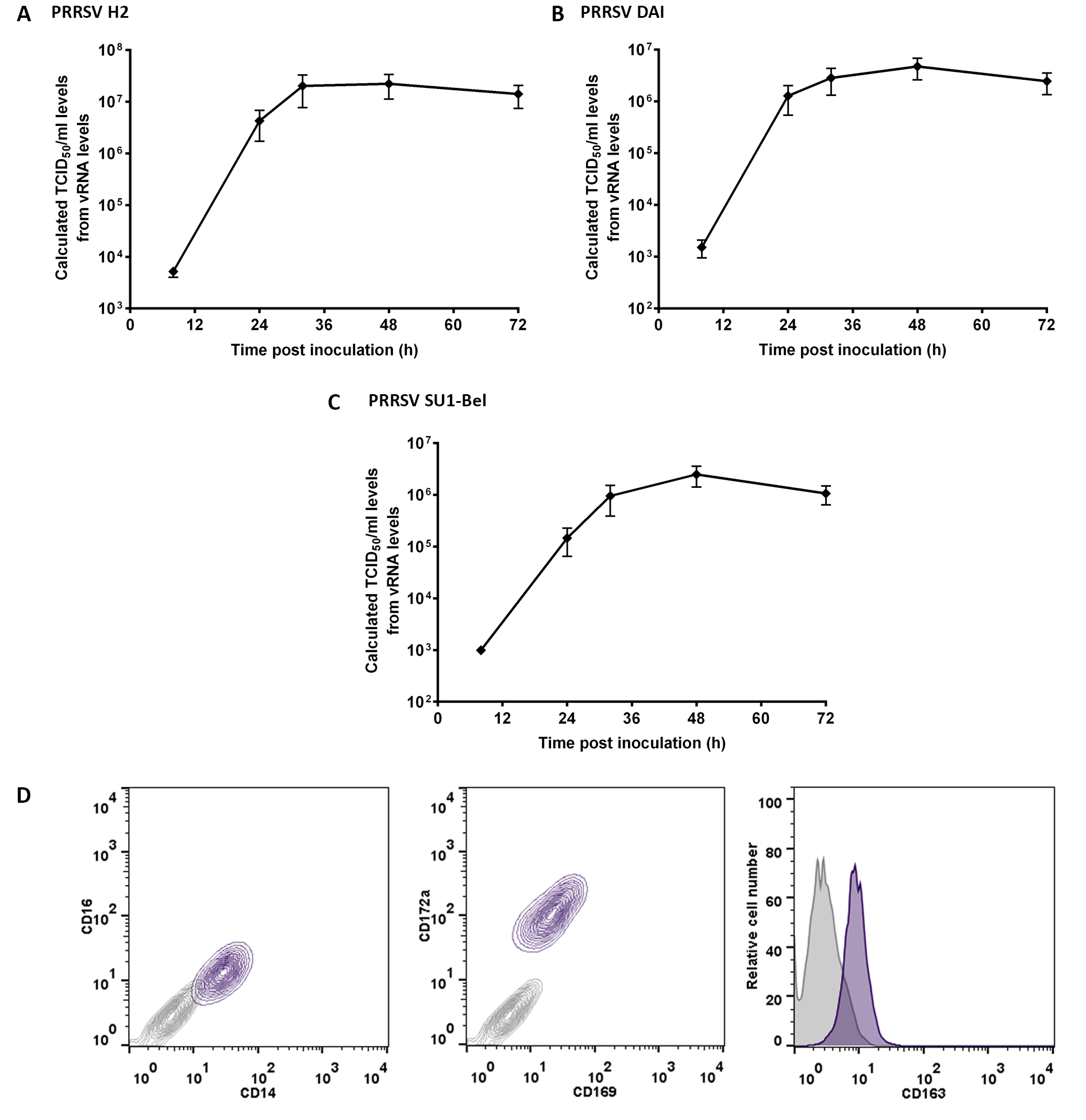

Supplement: S6 Fig — Peripheral blood monocytes were isolated from the blood of a warthog. Following cultivation in the presence of rhCSF1 for seven days PMMs were infected or analyzed by FACS. A-C) In vitro differentiated PMMs from warthog were inoculated at MOI = 0.1 of the respective PRRSV strain. Cell supernatant was collected at indicated time points to measure the released viral RNA by RT-qPCR. Error bars represent SEM, n = 3*2. D) Left; Co-staining with CD14-FITC and CD16-PE antibodies recognizing the native structure of the proteins (purple) relative to isotype controls (grey). Middle; Co-staining with CD169-FITC and CD172a-PE antibodies recognizing the native structure of the proteins (purple) relative to isotype controls (grey). Right; Staining against the native structure of surface expressed CD163 (purple) relative to an isotype control staining (grey). (TIF) [file ppat.1006206.s006.tif]

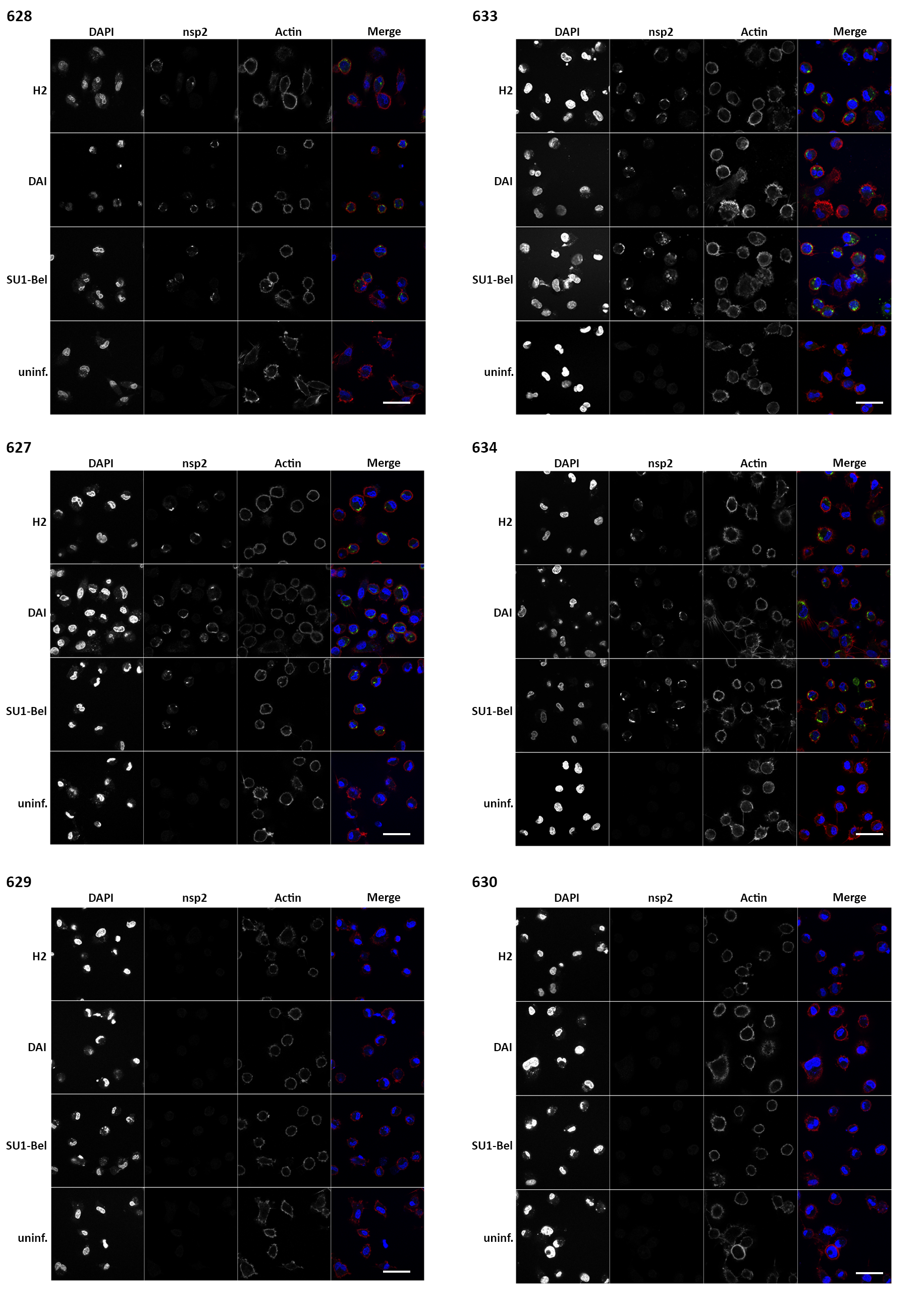

Supplement: S7 Fig — PAMs from wild-type (top panels), heterozygous (middle panels), and ΔSRCR5 (bottom panels) animals were inoculated at MOI = 2 with PRRSV genotype 1, subtype 1 (strain H2, top row), subtype 2 (strain DAI, middle row), and subtype 3 (strain SU1-Bel, bottom row). 22 hpi cells were fixed and stained with an anti PRRSV-nsp2 antibody, DAPI, and phalloidin. Scale bar represents 40 μm. (TIF) [file ppat.1006206.s007.tif]

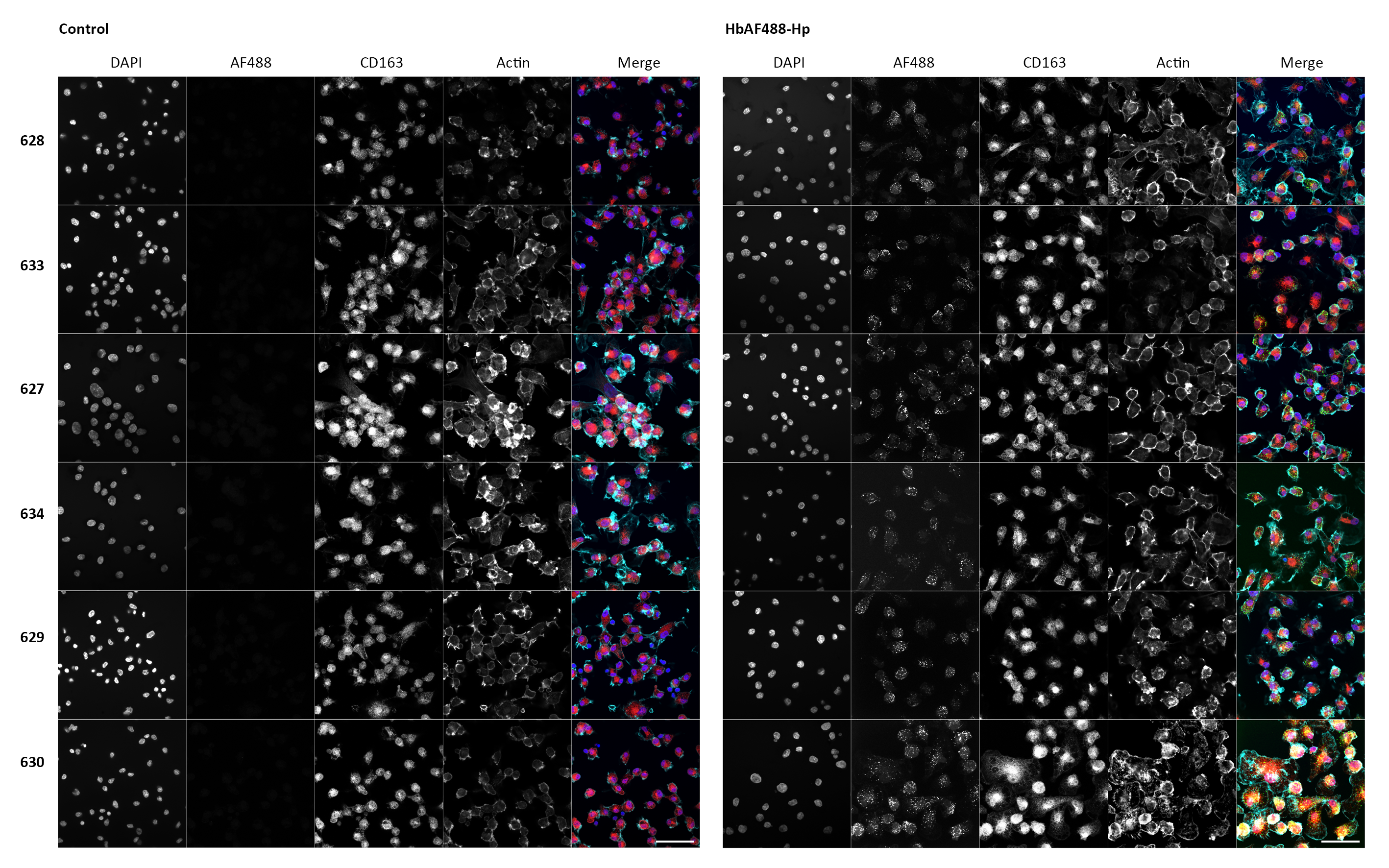

Supplement: S8 Fig — Visualization of Hb-Hp uptake. PMMs were incubated for 30 min with 10 μg/ml HbAF488-Hp. Cells were fixed, permeabilized and stained against CD163 and with DAPI. Scale bar represents 40 μm. (TIF) [file ppat.1006206.s008.tif]
