# Supplementary material for: Precision engineering for PRRSV resistance in pigs: Macrophages from genome edited pigs lacking CD163 SRCR5 domain are fully resistant to both PRRSV genotypes while maintaining biological function
Source: PLoS Pathog. 2017 Feb 23;13(2):e1006206. doi: 10.1371/journal.ppat.1006206 (PMC5322883; doi:10.1371/journal.ppat.1006206)
Supplement: S1 File — (PDF) [file ppat.1006206.s010.pdf]

Genomic sequence of the CD163 gene locus in large white pigs

Bold and green highlighted exons

sgRNAs and cutting sites are indicated in bold italic

1 TCTTCATCCT ATTAGAGACA CTGCTATACA GCAGAAATTG ACACAACATT GTAAATCAAC

61 TATACTTTAA TAAAATAAAA AAAAGAAATA CAAGTGCTTT CTACAGACAA TCTGCACAAG

121 TTATTTGTTA GACATATTTG ATTATAGAAT TAATATTAAA AGGGGTTATA ACAATCAAGC

181 ATTGATAATT TAATTATGTT TGCCTATTTT ACTTTAGTTT TTTGACATAA CTGTGTAACT

241 ATTGCGATTT TTTTATTCCT AATGTAATTA GTTCAAAACA AAGTGCAGAA ATTTAAAATA

301 TTCAATTCAA CAACAGTATA TAAGTCAATA TTCCCCCCTT AAATTTTTAC AAATCTTTAG

361 GGAGTGTTTC TCAATTTCTC AATTTCTTTG GTTGTTTCAT GTCCCATATG GAAGAAAACA

421 TGGGTGTGAA AGGGAAGCTT ACTCTTTTGA TTACTTCCCT TTTCTGGTTG ACTCCACCTC

481 CATTATGAAG CCTTTCTGTA TTTTTGTGGA AGTGAAATGA TTTTTAGAAT TCTTAGTGGT

541 TCTCTTCTTC AGGAGAACAT TTCTAGGTAA TAATACAAGA AGATTTAAAT GGCATAAAAC

601 CTTGGA**ATGG ACAAACTCAG AATGGTGCTA CATGAAAACT CTGGATCTGC AG**GTAAAATC
Exon 1

661 TTCTCATTTA TTCTATATTT ACCTTTTAAT AGAGTGTAGC AATATTCCGA CAGTCAATCA

721 ATCTGATTTA ATAGTGATTG GCATCTGGAG AAGAAGTAAC AGGGAAAAGG CAATAAGCTT

781 ATAAGGGGAA CTTTTATCTT CCATAGAATC AAAATTGAAG ACGTGACTAG AAGAAGGATT

841 AGATTTGGCA TCAGTTTTGT AAAATTGCTG AGGTGAAATT AAGTAAGGGA TGAAAATTAA

901 CTAAATTGTG TTGAGTATGA AACTAGTAGT TGTTAGAAAA GATAGAACAT GAAGGAATGA

961 ATATTGATTG AAAGTTGATG ACCTAGAGGA CATTTAGACT AACACCTCTG AGTGTCAAAG

1021 TCTAATTTAT GATTTACATC GATGCGTTAA ACTCATTTAA CATTCTTACT TTTTTCCCCT

1081 CAAGCATTTA AGCTGAAGTA TAACATTTCA CATGAAAGCC TGGATTATAA ATGCACAGTT

1141 CAGTGACCTA TCTCAGAGGA GTGACTGCCA TAGCATTTTT TTTGTCTTTT TGCCTTCAGA

1201 GCCACAGCAA CGCGGGATCC GAAGCCGCGT CTGCGACCCA CACCACAGCT CACGGCAATG

1261 CCGGATCTTT AACCCACTGA GCGAGGCCGG GGATCGAACC CGCAGTCTCA TGGTTCCTAG

1321 TAGGATTCGT TAACCACTGC GCCACGACGG GAACTCCTAC CATAGCATTT TTACTTTTAA

1381 GTTACTGTTG GTTTAGAGTA AGAAGGAGAA ATGAGAGTGA TGGAGCGTTT GCTATATTTG

1441 GAGACAAGGT CCTATATTGG AGGTTCTCAA ATATAAATTT TGTCGCTTTT TCCTCCAATG

1501 TATTGTTCAA CTACTATTTA GCAGGCCACT GTGCCAGGTA CTGGTGAAAC TGGTGAACAT

1561 GATAGATGTA ATTCATTCCC TCATGGAACT TTCCATCTAA CAATGTGGAT CAGGTAGGCT

1621 TGGAGATGAG AATGCCAGTG GTTGACTATG ACTCTGTGGC TGAAGGGAGA GCTACTCACT

1681 TCGTAGTTTC ATCAATGTCT TTTTGGTTTT CCAGGTTTTA AGCCCTGCTC TTGCAATTCT

1741 TTTCCCTTCT CCAACTTTCT TCTAATTTCT CACCCCTAGG ATGCCTATAA ACATGAGTAT

1801 TTTCAAAGCT ACTTCACTGA GGTTATATGA TCCTCGTGTG AATTTTTCCT GCCTGCCTTG

1861 CCATTTAGAA GGAAGTGTTT CCTGGAATTT CCATTGTGGC TTGGTGGTTA AAGACCCTGC

1921 ATTGTCTCTG TGAGGATGTG GGTTCAATCT CTGGCCTCAT TCAGTGAGTG GGTTAAGGAT

1981 CTGGTGTCGC TGCAAGCTGT GGCTAAGATC CCACATTGCC ATGGCTGTGG TGTAGACTGG

2041 CACCTGGAGC TCTGATTTGA CCACAATCCT AGGAACTTCA GATGTTGCCA TAAAAAGAAA

2101 AAAAAAGTTA GGAAGGGTTT TCTGTCTTGT TTTGACCTTT GTTAATCTCA AACCTTTGGA

2161 ACCATCTCTC CTCCAAAACC TCCTTTGGGT AAGACTGTAT GTTTGCCCTC TCTCTTCTTT

2221 TCGCAG**ACTT TAGAAGATGT TCTGCCCATT TAAGTTCCTT CACTTTTGCT GTAGTCGCTG**

Exon 2

2281 **TTCTCAGTGC CTGCTTGGTC ACTAGTTCTC TTG**GTGAGTA CTTTGACAAA TTTACTTGTA

2341 ACCTAGCCCA CTGTGACAAG AAACACTGAA AAGCAAATAA TTCTCCTGAA GTCTAGATAG

2401 CATCTAAAAA CATGCTTCAT GGTTTCAAAG GATCAGATAT TAAAAACCCC AAATAGGTAC

2461 AGAACCATGT GGCTCTCTCC CCCCAAACAA ATAAAACGTT AGCATGGTTT TCAAAAAAAT

2521 AAAATAACCT TCACAGGAAA AATGGATTTT ACTTAAGATT TGAAATAATA TCTAACTAAA

2581 AAATAGGGAA TAATGCAGAA GAGGAGAAAC CTCAGAATTG TTGGGATGAA GGAATTTTTA

2641 GTAACACTAA AAATTCAAGT GCCAAAATTT GTCTAAAATT GTATTCAGGG AAGCCAGATA

2701 TATATCAGTG AAATCGCCAG TTCCTATATT AGCTAAAATA ATCACAAGGC TGTAGCAGAG

2761 ACAGTTCAGA GAGAGGTGGA GATGAGATTT TTTTTTTTTA AGTATAATTG ATTTACAATG

2821 TTGTGGCAAT TTCTGTTGTA TAGCAAGAGA TAGAATTATT TTATGGTGGA AGATAATAGA

2881 AAAATATATC CATATCAATT TCCATTTGAG TAGATAAATT TCAATTAGAG TTCAACTAGC

2941 AATTAGTAGT TTTGCATACA TGGTGAAATA TATTCATGGT ATTTTGCATA TATGTGTGAA

3001 ATAGGTACTA AATTCCTCAT AACTGTTCTT TTTAGTCTCA CCATCAGCCT CTACTGATCT

3061 TAGGATTTTG GAGAAACATA CATAGTTCAT CCCTATAAAA TGCCATAAAA TCTCATTTTT

3121 ACATTAAACC ATCCAAGAGA TTATATAAAT TGACCTTATA AAGAATATCA GCCATAAAAT

3181 AAAGGTATCA TAGTATGGGA TTATTTAGCT TTATTGGTTC TATGTCACTG CTTAATTTGA

3241 AACCTGTGAT ATTGCTGTTT GTTTTTGAAC TCCTATGAAA TAACATTCTC CCATTGTACC

3301 ATGGATGGGT CCAGAAACAT TTCTCAAATC TGGCTTTGAA AAATAAATAA GTAATCTAAA

3361 GAATAATAAT TCTCTACTTG CTCTTTGAAT CTTGACCAAT TGCTGCATTT ACCTATTGTT

3421 ACAG**GAGGAA AAGACAAGGA GCTGAGGCTA ACGGGTGGTG AAAACAAGTG CTCTGGAAGA**

Exon 3

3481 **GTGGAGGTGA AAGTGCAGGA GGAGTGGGGA ACTGTGTGTA ATAATGGCTG GGACATGGAT**

3541 **GTGGTCTCTG TTGTTTGTAG GCAGCTGGGA TGTCCAACTG CTATCAAAGC CACTGGATGG**

3601 **GCTAATTTTA GTGCAGGTTC TGGACGCATT TGGATGGATC ATGTTTCTTG TCGAGGGAAT**

3661 **GAGTCAGCTC TCTGGGACTG CAAACATGAT GGATGGGGAA AGCATAACTG TACTCACCAA**

3721 **CAGGATGCTG GAGTAACCTG CTCAG**GTAAG ACATACACAA ATAAGTCAAG CCTATACATG

3781 AAATGCTTTG TGGGAAAAAA TGTATAGATG AGTTAAAAAC AAAAAGGAAC CAGTTTTCTA

3841 TAAGTCATCT AGTCCATGTA TAAAATTACC CAATCCATTA CTAAAAGACC ACTTCTGGTA
3901 TTTTACACAT GACAAAGCCC ATATTAAAAA AAAAAAATTC AGAAGAGATT CTGAATGCTA
3961 TAATAAATGA GCAAGTGACT AGCTTCAATT TTATATTAGG TCATTCTACC TTCTACTTCT
4021 ACATGAAAAT ATCATAATGT CTAAGTTAAT TCCTTGTCCC CTTTCCCAAT AAAGCACTGC
4081 TTTCATGCAC TGGCCTATGA ATCATGAACT TTTTGCCCTT TAACTGATGA TCAACTTACC
4141 AAATCAAGAA ATAAATATTC TTAGCACTGA TCCTTTTTTG TTGTTGTTGG AGGAAGAATG
4201 TTTTGCAAAG TAGAATTGCT TTTTTCTGTT TAACAGTGCT ATTCATTTCA TTTACATGGT
4261 CGTTTTAATT TATAAAACAT TTCATAAGTT TCACCTCATA TGCCCTTACA ATAACTCAGG
4321 AAGTTATATG TTAGACCTTT CTGCTGACAA ATCCCAGAGT CATGTTTCTG ACCCAGTTCA
4381 GATTCCTTGG CTTCCCATTT CTCTTTGCTC ATGTCATTGA CCTTTATGCA GCCCTCTTAC
4441 CTCCCACCTT TCTATTACAG ACCATCTCCT CCATAGGACT GGTGTTAGAA AGTACTAATC
4501 TCTACCCAGG CATTGTGGTG CAATGTGGGC AGCACAGGCT GGTATCTAGA AAAATGCTGA
4561 AGTGAATTCC AGCTCAGCTG CTCGTTAATA CTATTGTTTT AAGTAAGCTG TTCAATCCTT
4621 TGAAATTCAC TTTCTGAGCA CTCAGTGATA TAATAAATGT AGAGTTACTG GTACACTGTC
4681 TGGTATGTAA TAGGTGTTAA AAATTAACCT TAGTTTCCTC ATGGGTCACT GCTTCTCATT
4741 ACCTAGACAA CTCATTTCTC TTTCTTCCTC TTTCTCTTTC TCCATTCTCC TCCTCCTTCT
4801 TCCTCTTCTT CTTGTCTTTT ATTGTTATTC ATTTTGCTGA GAAAGTTAAG AAATAACAAC
4861 TCTAACCTCT ACATCGACCA CCTAGAGCAA AGTTAAAAAT AATAATAAAC CTTGCCAGAC
4921 TCTTACTATA ATTGTTGCTG TCTATAGAGT TGACTGTTTA AGTTAAGACA TCAGTATAGT
4981 ATTTTTAATT TTTGTGTTTT TTTTTTCATA CTTTTACATG AGGATCCTTT ATATAAGGAT
5041 GAGTTAAACA AACTTGATTT TTGAAGTTTA TACCCCTGAG GCTCAACTGC ATAATAATAG
5101 AAAGGGATCC ATAGCCTCTC AAGGACTTAA CTAGTTTCAT GAGTTTTCAG AATCTGAATT
5161 TCTGAGATTC TCCACCCCAA TTAAAGCTCA AGCCTCAGAA CATATATCCT TCTCTTGGTA
5221 AATTCTATTC TTATCACATG CGTAATAATA AAAAAGAGAG ATGTTGGAGA CAGATTTTTT
5281 TCCTCACATT CTGTCTCTAC TGTTTTCTAG GTGTTTGATT CTGTGTTATT TAACCTCAGT
5341 TTGCTTATCT GTGAAGTAGG GATTATGGTA ATAACATATA ATGCTTAATG TTGTAAAGAC
5401 TAAAGAAGAT AGCATATGTA ACACATTTGG AACAGGGAAT GCATATTTTG ATTGTGAGCT
5461 CTTATTATTA TTACCAATCA GCCATAATAA AAATCTTGTT AAGTGGAGGT CTTTGGATTT
5521 CAGAGCTTTT AAAATCTAAT TACTTTTTCA AAAAAGAGCT TCTTAGTGTT TTTTTTTTTT
5581 AACCACAAAG TGTTTCTATT TTTTAGGTGT CCCAAAATTT CATTCCAAAT ATCTTTTTCT
5641 CAGATATTTT AGTCCTCATA GAACACCTAG GGATAGTGTA TAGAGAAAAT TTTCTTTATT
5701 AAAAAGCTGT TCTTTGCTAA AAATTGTAGC AGGTACTTTT GGGAGGGGGG AAAACTTTGA
5761 TTCAGAAACT GCTAAGACAT GGAGTGTTTT GACTAATTTT TCCTCAATTT TTAATGTTTT
5821 TTATACCATA GGGTACTTTT GCAAACTATT ATGCATACTT ATATATTTTT ACTTTTTTCC

5881 TGTCTTTTAA CTTCCAAATT CAACTTCAGA CAATTATTCA TGCACTAAAC TGTTGTAGTA

5941 AGAAAGATTA AAATTAAAAA ATTAACCATT CAACAAATGA CTGGTTTGCC ATTTTTACTA

6001 CTTTGTTGTA TGAACAATTT TTTTTTCTAC AAATGAATAC TTTGAGTCTG ATTTATCCAT

6061 TCCTACATAA AAGTTTTTAC TATATCTTAG TATTGGAAGG AAACAAAACA AAACACAATG

6121 TAAATTTTAA TCTATAAATT TTGGGGGGGG GTAAATATAC ATAGATGAAA GTCTTAACCA

6181 TTAATTAGAG TCAAAAGATT AAAATTCTCC AATATGTGAA CTTAGGCTGC ATCCAAAATG

6241 AAGCATCATT TTTAAGGACA GCATCAAAAG TGACCAGAGG AATTTTACTT TCTTTCTTTT

6301 TTTTTTTTTT TTTGAATTTT AGTTTCTAAA CTCACTTCTG AATAAATACA ACTTCTAAAT

6361 TCTCGTCTTT TCTCTACTCT AG**ATGGATCT GATTTAGAGA TGAGGCTGGT GAATGGAGGA**

Exon 4

6421 **AACCGGTGCT TAGGAAGAAT AGAAGTCAAA TTTCAAGGAC GGTGGGGAAC AGTGTGTGAT**

6481 **GATAACTTCA ACATAAATCA TGCTTCTGTG GTTTGTAAAC AACTTGAATG TGGAAGTGCT**

6541 **GTCAGTTTCT CTGGTTCAGC TAATTTTGGA GAAGGTTCTG GACCAATCTG GTTTGATGAT**

6601 **CTTGTATGCA ATGGAAATGA GTCAGCTCTC TGGAACTGCA AACATGAAGG ATGGGGAAAG**

6661 **CACAATTGCG ATCATGCTGA GGATGCTGGA GTGATTTGCT TAA**GTAAGGA CTGACCTGGG

6721 TTTGTTCTGT TCTCCATGAG AGGGCAAAAA AAGGGGAGTA AAAGTCTTAA AAGCTCAAAC

6781 TGTTAAAAAC ATAATGATGA TTGCTTCTTT TATCATCTTA TTATTATCTA ATTTCAGGTC

6841 GAAATTCTAG TACCTGTGCA GTTTTTTACC TTAACTGAAA TTAAGATAAA TAGGATAGGG

6901 AGGAAGGATG AGCAGTGACA TTTAGGTCCA AGTCATGAGG TTAGAAGGAA ATGTTCAGAG

6961 AATAGCCCAT TCCCTCAGCC CTCAAAGAAA GAAAGAAAGA AAAAGAAAAA AAAAAAGAAA

7021 GCTTAACTAG AAAATTTTGT TCTCTGGATG TTTTAGAGGC AAACCATCCC TTTTATCATT

7081 CCTTACCTAC AAAGCCCTTC TCTTTAATCA CATTGACCCA CCCTTTCCTA AACTATTAGT

7141 TCAAATTCAC ATAATTGAAT GCTTTTAAAA CTTGGTTTCC TCTTATAATT ATATTTATGT

7201 TGTAAGGAGG CACTGTGTCT TGTCTAGAGA CTTTCATGTT CTATGCTTGA TTATGGGACA

7261 GGGACATGGC TTTGTCTGCT CCAGGATGTC ACTCTCCTTT TTTCACTTGA GCTCCTAGTT

7321 TGAAGAAGAC CTAGTAAGTC TTGAACTCCA GGGAGTCTTT AGGAAACTAT CCCTAGAGCA

7381 AAACTGTCCC TGAATTCACC CAGTGTCTTT TTTTTTTTTT TCAAATGAAG GAACTTTAGT

7441 TCAAACTAAA TTTAAAATAA GGGAATTCTA ATTCAGAATA CTGGGAAATC CAGGAGATTA

7501 CAATTGGCTT CATGTGTGAT TGGATTCAGC ACTTCACCAA TGTCATCAGG GTTCTGGTTC

7561 TTTTTTTATT TCTTGAATTG GCTTTTTTTT TTTTTTCCTT GTTGAACAAT ATGACTATCT

7621 ATACTTTGAA CCACAAAGAA AGTGATTCCT ACAGAAAAGA CAGAATGTGT TAGCTGAAGG

7681 AAGGGAATGG GACTTGGGGT AGAAAAAAAC ACCTTCCGTA TTCCTTAACC TATCAAAAAT

7741 TTCTAGGTAC CCCTAACTAA AATCCTAATT CAAGCATATT GGAGGAACTT GACAAATCCA

7801 GGAATAATAT TATCCGTTAT CAAATACATG CACATCATTT ACATTTCTCC ATGTCTCTGC

7861 TCATGCAGTT CCCGGCCCTA ACTCTACCAA AGTATTACTC TCCATCTCCC TCTTTTTTTT

7921 TTTAATGATT TTTATTTTTT CTGTTATGAC TGGTTTACAG TGTTCTGTCA ATTTTCTACT

7981 GTACAGCAAA GTGACCCAGT CACACATTCA TATATACATT CTTTTTCTCA CATTATCCTC

8041 CATCAGGCTC CATCACAAGT GACTAGACAT AGTTCCCAGA GCTATGCAGC AGGATCTCAT

8101 TGCTGCTCCA TTCCAAAGGC AACAGTTCAC ATCTATTAAC CCCAGATTCC CAGTCCACCC

8161 CACTCCCTTC CCCTCCCTCT TGGCAACCAC AAGTCTGTTC TCCAAGTTCA TGAGTTTATT

8221 TTCTGTGGAA AGTTTTATTT GTGCAGTATG TTAGATTCCA GATATAAGTG CTATCATATG

8281 GTATTTGTCC TTCTCTTTCT GACTGACTTC ACAAAGTATG AGAGTCTCTA GTTCCATCCA

8341 TGTTACTGCA AATGGCATTA TTAATCTCCA TCTTTTTTTG TTCATGTATA TGTTACCCAG

8401 ATTCCTTGAC TTTTCTACAT CATCAAGATA TTGTTGATCA CTTCTTTGTA GTGATTTCTG

8461 CCCTTCTCTG ATGTCCTGTG ACACTAGTCT GGATTATTCA TTTACCTGAA ACCACATGTC

8521 TCTTATAATG TGTATCCCAA ATTAAATATG TCTATTGTAA TGTGTATCCC AAATTAAATA

8581 TTTATCTTTC TAAAAAAAAA AATTTCTAGG CCCCCAATCA GCATGTTTCT TCTCAGTGTG

8641 TTTTATACAT GCTGCAGAAT CATAATAGAC AGCATAATAG ACAGCATAAC AAAAACTAAA

8701 AATGCCAGGG GAAAAAAGCA ATTTACTGAT TACAACATAT TACTCAGAAT CAAGTTCTGT

8761 TCTTTGAGGA ATATTGATTG GGGGAAAATG AAAATAATGA TGGGGAGGTC CCTTTTCTCT

8821 TTGCTTTGCT TTTAAACTAC GGAAGTAGTC AGAAAGGGGT CAGGAATGTA ATATAAACCA

8881 GGTAGTCCTG GTAGGTAACG CAGCCGGAGG CAAAAGTGAG TGTTGAGTAT TGAGGCAAAC

8941 TGGAGGGCAT GGATACCACC TAGACAGATG CAAATATATA TTTAACAGGG AAAAAAGAAC

9001 CAAACAATTT CAACAAAAAA CCAAACAATT CCAACAAAAT TGGTCCAATA AGCAAACCTC

9061 TAGATAAATT TCAGTCCCTG GATGTTTTGT TAGGAACTCT TCCTACAATG CGTGCTTTCC

9121 ATTCTGAAAA GTCCTATCTA CTTGCCTGAT CCACTTCTCC TTCCATCCTA AACGATTTTC

9181 AGTGGTAGTA TATTACTGTT GTCTCTGTCT CTACTTATAT ATCTTCCCCT TTTCACTCAC

9241 TCCTCTCAGG TACAGCTCTT CAGTTTGCCC TTATTCTTGT TTCCTTGTCA ATGACTTGTT

9301 TTGTGTCCCT CTTACAG**ATG GAGCAGACCT GAAACTGAGA GTGGTAGATG GAGTCACTGA**

Exon 5

9361 **ATGTTCAGGA AGATTGGAAG TGAAATTCCA AGGAGAATGG GGAACAATCT GTGATGATGG**

9421 **CTGGGATAGT GATGATGCCG CTGTGGCATG TAAGCAACTG GGATGTCCAA CTGCTGTCAC**

9481 **TGCCATTGGT CGAGTTAACG CCAGTGAGGG AACTGGACAC ATTTGGCTTG ACAGTGTTTC**

9541 **TTGCCATGGA CACGAGTCTG CTCTCTGGCA GTGTAGACAC CATGAATGGG GAAAGCATTA**

9601 **TTGCAATCAT AATGAAGATG CTGGTGTGAC ATGTTCTG**GT AAGTGAAAAC AAAACACCGG

9661 AAGGACCTGT GTTCTTCAGG ATTAGGAATG GATATGAGAT AGGAGAAAAA TTGTATCTAA

9721 TATTTTCTTT GTTGGGAATT CTTTTACAGT TGTGACAAAT CTTTAACATA TTCTTCATTT

9781 GAGTAGTTTG GAGGGTTGTC TGACTGTTTT CTATAATAAA TGTCCCAAGT GCTATGAGGT

9841 ACCACATTTC AAATTCTAAT TCTACCTGAA GCTCCAAAAA GACAAAATGT TATAGGTCTT

9901 TTCTTTATAT CTAATTTGCT TATGGTTTTT AGCCATTGAC AATTTTTTTT TTCTTAACTC

9961 TTGAAACTAT AATCCTATTT CTAACCAAAT TCATGTTCTA TACTGGCTCT TCAAAAACCC

10021 AGGAGATGGG AAAGCCAGAA TCTCCAGTGT TTCAGCTTCT GGGAAGGAGC AAGTTTTTAA

10081 ATGTGGGAGC TAAATTCCAC ATGTATCTAT GGCCTAAGTG TATGTTTATT TTGCAG**ATGG**

10141 **ATCAGATCTG GAACTGAGAC TTAAAGGTGG AGGCAGCCAC TGTGCTGGGA CAGTGGAGGT**

Exon 6

10201 **GGAAATTCAG AAACTGGTAG GAAAAGTGTG TGATAGAAGC TGGGGACTGA AAGAAGCTGA**

10261 **TGTGGTTTGC AGGCAGCTGG GATGTGGATC TGCACTCAAA ACATCATATC AAGTTTATTC**

10321 **CAAAACCAAG GCAACAAACA CATGGCTGTT TGTAAGCAGC TGTAATGGAA ATGAAACTTC**

10381 **TCTTTGGGAC TGCAAGAATT GGCAGTGGGG TGGACTTAGT TGTGATCACT ATGACGAAGC**

10441 **CAAAATTACC TGCTCAG**GTA AGAATTTCAA TCAATGTGTT AGGAAATTGC ATTCTACTTT

10501 CTTTTACATG TAGCTGTCCA GTTTTCCCAG CACCACTTGT TGAAGAGACT GTCTTTTCTT

10561 CATCATATAG TCCTACATCC TTTGTCATAA ATTAATTGAC CATAGGTGTG TGGGTTTATA

10621 TCTGGGCTCT CTATTCTGTT CCTTTGATCT ATGTGTCTGT TTTTATGCCA GCACCATGCT

10681 GTTTTGATTA CTATAGCTTT GTAGTATCAT CTGAAGTCAG GAAACATGAT TCCTCCAGCT

10741 TTGTTCTTCT TTCTCAAGAT TGTTTTGTCT ATTCAGAGTT TTATGTTCCT ATGCAGATTT

10801 TATTTTTATT TTTATTTTAT TTTTATTTTT TTTATTTTCC CACTGTACGG CAAGGGGGTC

10861 AGGTTATCCT TACATGTATA CATTACAATT ACAGTTTTTC CCCCACCCTT TCTTCTGTTG

10921 CAACATGAGT ATCTAGACAA AGTTCTCAAT GCTATTCAGC AGGATCTCCT TGTAAATCTA

10981 TTCTAAGTTG TGTCTGATAA GCCCAAGCTC CCGATCCCTC CCACTCCCTC CCCCTCCCAT

11041 CAGGCAGCCA CAAGTCTCTT CTCCAAGTCC ATGATTTTCT TTTCTGAGGA GATGTTCATT

11101 TGTGCTGGAT ATTAGATTCC AGTTATAAGG GATATCATAT GGTATTTGTC TTTGTCTTTC

11161 TGGCTCATTT CACTCAGGAT GAGATTCTCT AGTTCCATCC ATGTTGCTGC AAATGGCATT

11221 ATGTCATTCT TTTTTATGGC TGAGTAGTAT TCCATTGTGT ATATATACCA CCTCTTCTGA

11281 ATCCAATCCT CTGTCGATGG ACATTTGGGT TGTTTCCATG TCCTGGCTAT TGTGAATAGT

11341 GCTGCAATGA ACATGCGGGT GCACGTGTCT CTTTTAAGTA GAGCTTTGTC CGGATAGATG

11401 CCCAAGAGTG GGATTGCAGG GTCATATGGA AGTTCTATGT ATAGATTTCT AAGGTATCTC

11461 CAAACTGTCC TCCATAGTGG CTGTACCAGT TTACATTCCC AGCAGCAGTG CAGGAGGGTT

11521 CCCTTTTCTC CACAGCCCCT CCAGCACTTG TTATTTGTGG ATTTATTAAT GATGGCCATT

11581 CTGACTGGTG TGAGGTGGTA TCTCATGGTA GTTTTGATTT GCATTTCTCT TATAATCAGC

11641 GATGTTGAGC ATTTTTTCAT GTGTTTGCTG GCCATCTGTG TATCTTCTTT GGAGAAATGT

11701 CTATTCAGGT CTTTTGCCCA TTTTTCCATT GATTGATTGT TTTTTTTGCT GTTGAGTTGT

11761 ATAAGTTGCT TATATATTCT AGAGATTAAG CCCTTGTCAG TTGCACCTAT GCAGATTTTA

11821 AAACTATTTT CTCTAGTTCT ATGAAAAATA CCATTGGTAA TTTGATAGGG ATTGCCCTGA

11881 ATCTGTAGAT TGCCTTGGAT AGTATTGCCA TTTTAACAAT ACTGAATCTT CCAATTCGAG

11941 AGCACAGTGT ATCTTTCTTT CTGTGTCATC TTCAGTTCTT CTCATCTGCA TCTTATAGTT

12001 TTAGAAGTAC AGGTCTTTTG CCTCCTAAGG TGGGTTTTTT CCTAGGCATT TTATTCTTTT

12061 CAATGTGATA GTGAATGAAA TTGTTTCCTT AATTCTTTCT CTCTCTTTTT TAATGGCTTC

12121 ACCTGCAGCA TATGGAAGTC CCCAGGCTAG GGATCAAATC ACAGCTGCAG CTATGTCCAT

12181 GCCACTGCCT TGGCAACAGC AGATCTGAGC CACATCTGCC ACTTACACTG TAGCTTACAA

12241 TAATGCTGAA TCCTTAACCC ACTGCTAGAA CCTGAATCCT CACAGAAACA ATGTCGGGGT

12301 CCTTACCTCT CTGAGCCACA ATGGGAAATC TTCATTTTTC TTTCTGATAA TTTGTTGTTA

12361 GTGTATAGAA ATGAAACAGG TTTCAGCATA TTAATTCTTA TCCTGAAGTT TTACCCAATT

12421 CATTGATAAA CTCTAGTAGC TTTTTGGTGG TGTCTTTAGG ATTTTCTATG TATAGATTCA

12481 TGTTACCTGC AAACAGTGCC ATTATTACTT CCTTTTTTCC AAATTGGATT CCTTTTATTT

12541 CTTTTTCTTC TCTGCTGTGA CTAGGATTTC CAAAATCATG TTGAATAAAA GTAGCAAGAA

12601 TCAGCATCCT TGCTTTGTTC CTGACCTTAG AAGAAACACA TTCAGCATTT AACTGTCGAG

12661 TATGATGTTA GCTGTGGGCT TATCATATAT GGCATTTATT ATTTTGAGGT ATATTCCCTC

12721 TATACCCACT TTGTTGAGAA CTTTTTATCA TGAATGGATG TTAAACTTTG TCTAAAGCTT

12781 TTTCTGCATC TAGATAACCC TATTATTTTT CTTTTCTAAT TTGTTCATGT GGTGTATCAC

12841 ACTGATTTAT TTGCAGATGT GCATCCATTC ATGTATCCCA CTTGATCGTG GTGTGTAATC

12901 TTTTTAGTGT ATTAGTGAAT TTGGTTGCTA GTATTTTGTT TGAGGATTTT TGCATATACA

12961 TTCATCAGCG GTATTGGATT TTAAATCTTT TGTATGTGTC TTGTTTTGGT ATCAGGGTAT

13021 CCTCTAGGGT ATCCTCCTAG AATGAGTTCA GAAGGGTACA TTTCTTTGGG GAATATATTT

13081 GGTAGAATTC ACTTTTGAAG CTGTCTGGTC CTGTTCTTTT GTTTGTCGGG AAGTTCTTTT

13141 TAAATTATTA TTATTACTGA TTCAATTTCA TTACTGGTAA TTGGACCATT TATATTTTCT

13201 TTTTTTTCCT GGTTCAATCT TGGGAGATTG TATGTTTTAA AAATTTGTCC AGTTCTTCTA

13261 GGTTGTTCAT TTTATTGGAA TGTAATTGTT TGTTTATCTT TTTTTTTGCA TTTTCTAGGG

13321 CCGCACCCAT GGCATATGGA AGTTCCCAGG CTAGGGGTCT AATCGGAACT GTAGCCACTG

13381 GCCTACCCCA GAGCCACAGC AACGTGGGAT CTGAGCCGCA TCTTCGACCT ATACCACAGC

13441 TCACAACAAT GCGGGATCCT TAACCCACTG AGCAAGGCCA GGGATTGAAC CTGCAACCTC

13501 ATGGTTCCTA GTTGGATTAG TTAACCACTG AGCCACGACG GGAACTCCAA TGGTATGTAA

13561 TTGTTTATAG TGATCTCTTA TGAGTCTTTA TTTTTCTGTA GTAATCATAA CTTCTCTTAT

13621 TTCATTTTGA TCTTATTGAC TTGAGCCCTC TGTTTTTTTC TTAGTGACTC TAGCTAAAGG

13681 TTTATCAATT TTGTTCATTT TTTTCAAGGA TCTGGCTCTT AATTTCATTC AACTTTTCTA

13741 TTTATTTTAG TCTCTATTTC ATTTACTTCT GTTCAGATTT TTATGATTTC TTTCTTTCTA

13801 CTAAGTTCAG TTTTGGTTTG TTCTTTTCTA TTTCCTTTAA GTGTAAGGTT ATGTTGTTTA

13861 TTTGAGATTT TTGTTTCTTG AGGAAACAGG CTTGCATATT TGTAAACTTC CCTCTTAGAA

13921 TAGTTTTTCT TAAGTTCCAT AGTTTTTTTT TTTTATTTTG TGGTTTTTAT TTTTCCATTA

13981 TAGTTCATTT ACAGTGTTCT GCCAATTCCT ACTATATAGC AAAGTGACCC AGTCATATAT

14041 ATATGTATAT ATGTATATAT ACACATACAT ATACACATTA TCCTCCATCA TGTTCCATCA

14101 CAAGTGACTG GATACAGTTC CCTGTGCTAT ATAGCAGGAT CTCATTGCTT ATCCACTCCA

14161 AATGTAATAG TTTGCATCTA TTAACCCCAG ATGTCCCATA GATTTGGAAT TGTGTTTTTG

14221 TTTTCATTCG TATTCAGGTT TTTTTTAATT TCCTCTTTGA TTTCTTCAGT AATCCATTTG

14281 TTGCTTAGTA ATATATTGTT TAGCCTCTGC GTGTTTGTGG TTTGTTGCAA TTTTCTTCTT

14341 GTAGTTGATT TCTAGTCTCT TTGTGTTGTA GTTGGAAAAG ATGTATGATA TGATTTCAAC

14401 TTTCCTAAAT TTACCAAGGC TTGTTTTGTG GCCTAGCATG TGATATATCC TGAAGAATGT

14461 TCCATGTGCA CATGAAAAAA ATGAATATTC TGCTGCTTTC AAATGGAATG CTCTCTCTAT

14521 TTCAATTATG TCCATCTCTA ATGTTTTGGG AACATGTTCT TTTGCTACCT CATTTTGCCT

14581 AATTTGCTGT TTTGGGTTCT AAATATCTGG TAGGTTGGTT ACATTTTCCA ACCTTGGACA

14641 AATAACCTTT TGTTGAAACA TCCTGTGCTT CCCAGCAGCA CACTCCTCTC TGGTCACCAG

14701 AGCTATATGT TCCAGGGGTG CCCCCCTATG CTGACTTTGT GAGAACTTCT TTTGCAGTTG

14761 GCTGACTACT GTAGGTGGTC TTGTAGGCAT GGCTGGCCCC CAGTCTGGTT GTTTGCAAGA

14821 AGCTGCCTTG TACAAAGGCT GCCAGTCACT TGTTGGTGGG ACTGGGTCAT GGGGTGGCTG

14881 GCTATAGAGA CCAGGGTTGT CTCAGGGGTA GTGCTGTCTC ATTTGTGGGT TTAGCCACGT

14941 TTTGCAGTGG GTGATTGTGG TTCCAGGGTT CCTAGATCTA GTGTCAGCTT GTGGGTACTG

15001 GGGTCCCCAG CTGCAGGGCC TAGGAGCTTC AGAGCTAGAG CTAACCTCCT GGTGGGTAGA

15061 CTGTGTCCTG ACAAGGCAGG TTGTAGTGTT ACAGTGATCC TGGGGCTAGT ATCTATCCAC

15121 TGGGGGGTAA GACTTGTCCC AGGGCTAGCA CCAGCTCTCT GGTGGGTAGA TCTAGGTCCT

15181 GGAGGTTCTG GCTGCAGGGC CAGGGATCCA GGAGCTGGTG TTGACTGGTT GGTGGACAGG

15241 GCCAAGGCCC AGAGTGTCCC CAGGCTAGAT CTACTTCAGT GATGGGTGGA TCTAGGTCCT

15301 GTATTTCTGG CTACAGGGCT CTGGGATCCC AGAGTTGGTA TGTCAGTCAA CTGACATACA

15361 GGGCTGGAGG CAGAGAGTCC TGAGGCTGGT GCCTGCCCAC TGGTGGGTGG AGCTGGGATT

15421 CAGGGTCTCT GACTGAAGTG CCCTGGGGAT CCCTGGGCTA GTGCTGGCCC ACTGGTGTGT

15481 GTTTGGTTGG GTCCTGGCCA TTCTGGTAGA CAGGGCCATA TTCCCATATT CCAGGGTGGC

15541 TGTAGGCTCA GGGAATCTCA AGGCAACCTA CTGCTGGTTA GAGGAGTGTG TGGGGAGGTG

15601 CTATGTCCCT GTCCAGTTTG TTGCTTGGCA TGAAGCATCC CAGTACTGGT GCCAACAGGC

15661 TAATTAGTGG GTCTGGGTCC TGGTGCTAAT AAGCTAGAGG GAAGATTCAA AAATGACATT

15721 TTTTTAACAC CAGTGTCCTT GTGGTAAAAT GAACTCCCCA GAATGGCTAC CACCAGTGTC

15781 TATGTCCCCA TGGTGAATTC TAATTGCTCC TGTCTCTTGA AGTGGCTCTC CAAGATCAAC

15841 AGGTGGGTCT GATCTAAGCT CCTTTCAAAT TACTGCTTCT GCCCTGGGTC CCAGAACATG

15901 TGAGATTTTG TGTGTCCTTT AAGAGTGGAG TCTCTATTTC CCACTGCTCT CTGGTTCTCC

15961 CCAAAGTAAG CCCTGCTGGC TTTCAAAACT TCTGGGAGCT TGCCTTCTTG GTATAGGACT

16021 CCTGGGCTAG GGAGTCTAAT GTTTGGCTTA GACCCCTTAC TGCTTGGGAA GAATCTCTGC

16081 AACTGTAATG AATTATCTTC CTATTTGTGG GTTGCTGAGG ATATGGTCTT AACTGTTCTG
16141 TGTTCTACCC CTCCTATCCA TCTTGTTGTG GTTCCTTCTT TATATCTTTA GTTGTAGAAA
16201 AGTTTTTCTT ATCAACAGTT GCTCTGTAAA TTGTAACTTG GGTGTACACC TAGTAGGAGG
16261 TGAGCTCAGG GTCTTCCTAC TCTGCCATCT TGGCCATGTC CTCTAAACAT TTTGGTGTAT
16321 TTCACTGCAA CCTTTTTAAA AATCTCAAAA GTGAGCTGTG ATTGGCTAGT CTTGTGGATA
16381 ATCTCTAGCA TTTGATGCTA ATCATATTTA TACAAATACT TTGTTGAAAA GTGATGCCTT
16441 TTTAACTATT ATTAAAAAAC GTATTGACAT AACTATTGCT ATTATACTGA AAAGAAAGAC
16501 CTTAGAGAAA ATAGCATAAG AGCAAAACCA TTAAACATGG AGACATCTAG TCATAGGGTG
16561 GAAATTTTAT GTGGTGCATA TCCCCTAACC AGTGGCTTTA CACCAGGCAC ATCCTAACTA
16621 AGATCTGCTC CCAAGTGTCT TCCCTGATGC TTTAAATTGT GTTACATGGA AACTATCCTT
16681 TGATGAAGAA ATGCAACCTT TTAAAATACA ACATTGAAAC TTTTGTGCTT TAATTTTGCT
16741 TTTCAACATT TTTTCTTTTT AAAAGAAGAA ATTTATTTGT TTTTTTAAAT TTTAATGGCC
16801 ACGGCATATG GAAGTTCTCA GGCCAGGGAT AGAATTCAAG CCACAGGTGC GACCCATGCC
16861 ACAACTGCTG CAACACCAGA TCCTTTAACC CACTGCACCA GGCCAGGGAT TGAAGCCTTG
16921 CCTTACTGAC AATCTGAGCC ACTTCAGTCA GATAAAGAAA TTTCTTCATT AAGCAGAGTA
16981 TTCACATGGT TTAAACTTCA AAATATTAAA GTGTAAACTC TTTCCCCACC ACTGTCCCCA
17041 GCTCACCAAC TCTACTTACC ACAGACAACT GATGTGGTTA GGGTATTTAA ATAGTAAATC
17101 CAAGAAAATA TAAACAAATC CGTATATATA GGTTTCACCC CATTTTATTA TCCTAATGTT
17161 GCATATCATA TAAACTATAC TGTCCCTTGG GTATTCACTT AGTAAAATAT TTTGATCATA
17221 ATTTCCTATC AGTATTTAAA GAGCTTTCTG AAATTATTTC TGTATAACAT TTCTTTTCTC
17281 ATCATCTATT ATGTGCATTT ATTTATATTT TAACTTCTTT TATTAGATGA AATTATCTTC
17341 TGCTTCAGCT TTTTTTTTTT TTTAAGAACA CACAGTTGGG TTTTTTAAGG TTAATACCAC
17401 CTTTGTTTTC TAAGTCATTA AATTTGTTTT TCTATTAATT CACTTCTGAT TCTTTGAAGT
17461 TTGATTTCTT TTTAGCTTTT AACTTCTTGA GTTGTATGCT TAATTAATTT TGATTCTTTC
17521 CTATTTATTA ATATACATAT TTGAAGCTAT AGGTTTTCCA CTGAGTATAC CAGTAGCTAT
17581 ATCGTATAAT TGATGAACTG ATCCTCTGTG AGTCTGGGAC ATAAACGTCC TATGACTGTT
17641 ATGTGGTAGC TGTGAATTGC TCTTTTTAGA TTATAAAGTT CTCATCTTTT ATAGTTGAAC
17701 AATTTTTGTC CTGAATCAAA TTTGTTGGAT ATTAATATCA CATCTATTGC TTTATTTATT
17761 TTCTATTCTC ACTTTTAACC TCTGTGAATA ATTTCACTCT AGGTGCCTCA CTTTTTTCAT
17821 AATAGAATTG GGATTTATTT TTAAAAGGAC TCTGATTAAG TAATTTTCTT TTTCTGATAT
17881 GGGAGATATA TTTGACCTTA ACTTAGTCAC ATTATGCATT GTTCTCTTGT CATGTTATGT
17941 ATACATAACA TTTATTGTCA TTATGGTACA ACTAAAAACA TATTTCACTC TGTGACCTTT
18001 ATGGGGACTC AGCATTTGTT TAGGAATGTG GAAGTATATT TGTATATCTG ATAATTTCCT
18061 TCCAAATTTA AAAAGGTTTG TATATTTTCA TATTAACATA TTTCATATTA ATTAGCATGA

18121 ATTTCAGCTG CATTAAAAGG AAAACCACCT GAGTGGTAAA GAAAAAGTTT TTTTTTCTCT
18181 TTTTTTTTTT TTTTTTTTTA ATGGCCACAT CTGTGGCATG TGAAGTTCCC AGGCTAGGGG
18241 KCGAATAGGA GCTACAGCTG CCAGCTTGCA CCACAGCCAC AACAATGCCA GAGCCAAGCC
18301 TCATCTGCGA CCTATACCAC AACTCATGGC AATGCTGGTT CCTTAACCCC CTGAGTGAGG
18361 CCTGGGGTCA AACCCACATC CTCATGGATA CTAACCGGCT TTGTTACCGC TGAGCCATGA
18421 GGGAAACTCC CTTTTTCTCA TTGAAAATAA GTCAAATAGA TAAGCAGCTT AAGGCTGTTT
18481 GGGTGATTCT GTGGTCCAGT AATTATCAAA TCCTACTGGA CAAGAATAGA GAATGTGCAA
18541 ATGAGGGAAC GTGTTGGTGA GATCAGGCTC TGCCCACTGA GCTATCCTCT GTCATGGGCC
18601 CTGTGCTGTT CTCAGAGCTG TACTTCCTAG GGCATTGTTC TCATTTCAAT TCTGAGTTCA
18661 GTGTGGAGAG TATACGTGTG TGGGGGCTGC ACGCTTTTCA CAACCCACTT TCTGCTGATA
18721 CTGATTTAGG GATCCTTGGA TTGCTTTACA GTTGAGTCAT CATTAACTAG TGTCACTTGC
18781 CTTCAAAGTC AGCAAAATAA TTGTCTCCAA ACTAGTAGGC TTCTAGTGTA TTTGCTTTAA
18841 TCCAATGCCA TGTGAAAGTA ACATGGTCAA AGAATAAGTT ATATACCTTG ACCTACCCTG
18901 TGACCAGGCT CTTCCTCTTA ATTTATTGAC CACTGCCTTA AGGTCATTTG AAACCATGGG
18961 TTTGGGAGGA AGGCAAGGCC TAAATCCCGT CTTTGTTGGA AGGCTCACTG TCCTTGTCTT
19021 TAGAGCATCA TTTTTTTTTA AACTGGGGTA CAGTTTATTT ACAGTGTTGT GTCAATTTCT
19081 GCTGTACAGC ATAGTGACCC AGTCATACAC ATACATACAT TCTTTTTCTC ATACTATCTT
19141 CAATTTTATT TTGTGCTAAG TCTGCCATTT TATCATCACC TCAGTTTGAA GGACAGGATA
19201 TTTAGAGTTT GTTTTTTTTT TCCCCCCAAT CCTGCAATTT CTAAATTATA AGACTCTCAA
19261 TTAGCCGTAT ATAACAGCTG CAGGCACAGG ATGTCTCCCT CACAAAATTG GTATTTTTCC
19321 TTCCATTTCT TCTTGCAGTT TGGCTATTTC TTGTCTGAGT TCATCTCTCT TTTTAAGTGT
19381 TAAAAAGGGC AAGGAGGATT CATGCTATGT CAACATTATG ATTTTTTCTT TTCTATACTT
19441 GATAAGAGTA TACTTTTCCC AAATGTCATC CAACTTTTCA GCATCAGTTT GGACATGGTT
19501 TTCTTTTCAA GGTGGTATTT CTCTAATGTC ACTTGAATAA CAAGACTCGT TAGTTCTCCA
19561 GGCTACAATA TCCTAGTCTG AGTATATTCT GCATGTTAAT TCTATTCAGC CACATCCATA
19621 ATTTAGGTTT TATTCCTGGA ACACCTCACT TTTTTTTTTT TTTTGGTCTT TTTATAGCCA
19681 TAACCATGGC ATATGGAGGT TCCCAGGCTA GGGGTCTAAT CTGAGCTTTA GCCACTGGCC
19741 CATGCCACAG CCACAGCCAT GCCACATCTG AGCCACATCT GTGACCTTTT CCACAGCTCA
19801 CAGAAACACC AGATCCCTAA CCCACTGAGT GAGGCCAGGG GTCAAACCTG TAACCTCATG
19861 GTTCCTAGTC AGATTCGTTT CCTCTGTACC ACGATGGGAA TTCCTAATAC CTCACTTATG
19921 ATAACACATT CTGAATTATT TAGGATTCTA TTATACTGCA TGTAATAGAA ATCCCAAATA
19981 GCAAAATTTG CAACTTAAGG CAGGTTCCTG TCTTTACAAA ATCATGTTTT CCTTTGCTAT
20041 ATGTGCACTT TGCTTTCCTC TGTGAATTCC CTTTTTTGTT ATATTTCTAT AGCTTTTGGA
20101 AACACTTTTA CTTATTTGGG GGGGCCTAGA TTTTTAACCC TCTCCTTGTT TTTCTAGAAA

20161 TAGAGTTTAT AATTTTATTT CTTCATTTAC TTGATACTTT CAAGAGATTT CCAGGAAAAA
20221 AATTATGGAA ATACTGTCTC TGTGCCTGCC AAGTTCAAAC TAAGAATTGT ATAATCTGTT
20281 TTAATTCTTA AGCATTTATA GATGACAAGG CTTTGTGTCT GATAGGGGCC AGCGAACTCA
20341 GTAAAGAGGG AAGATGAGAA AGATAATGGC AAGAATTTAT CCCTGAAGTG TAGTTTTGAC
20401 AAACCAGTCA CAAAGAGGTC TAAGAAATTT TGGTCACAAA GTTGTTTTGA ATCCCAGGCA
20461 TTTTATTTGC AATGATTGCA TATGTTCTGG AAAGGACATC TGAACCTAAG AAATAGTTCA
20521 TTTGCATTGT GTTATATTTT ACTAAGGTCT GAGAAATAAT CTTGAGATGA GAATGAACTC
20581 TACTTCTTCA GAGTCTGGAA GGAATAAATT ATGAAAATGT ATTAATGCTT CTTTAAACCA
20641 TATTGTATAT TTATCTATTA CTAAACAAAA AGAAGTAGCT CTATTTATTT ATTTATTTAT
20701 TTATTTATTT ATGTCTTTTG TCTCTTTAGG GCCACACCTG TGGCATATGG AGGTTCCCAG
20761 GCTAGAGGTC CAATTGGAGA TGTAGCAGCC AGCCTATGCC AGAGCCACCG CAACACGGGA
20821 TCTGAGCCAC GTCTGTGACT TACACCACAG CTCACAGCAA CGCCTGATCC TCAACCCACT
20881 GAGCGAGGCC AGGGATCGAA CCCATGTCCT CATGGATGCT AGTTGGGTTC ATTAACTGCT
20941 GAGCCATGAT GGGAACTCCA AATTAATTAT TTCTTATATT TGTTCTTCAT ATATTCATTT
21001 CTATAGAAAG AAATAAATAC AGATTCAGTT AATGATGGCA GGTAAAAGCT TAACTTATTA
21061 ATCAAAGGAG TTAATCCAGG CACAAAAATT CAATTCATGG CTCTCTGTTA AAATTTAGGT
21121 ATAGGTTTAG CAGGAAGAAA AGGTTAGTAG ATGCAGACTA TTACATTTAG AATGGATGGA
21181 CAATGAAGTC CTACTATACA GCACAGGGAA CTATATCCAA TCTCTTGGGA TAGAATATGA
21241 TGGAAGACAA AATCAGAACA AGAGAGTATA TATATATGTG TGTGTGTGTG TGTGTGTGTG
21301 TGTGTGTGTG TGTGTGTGTG ACTGGGTCAC CCTGCGGCAC AGCAGAAATT GGCAGAACAT
21361 TGTAAATCAA CTATACTTTA ATAGGAAAAA TACTTTTAAG GGCTAAATTT CCAATATTCT
21421 AACCATGTAC ACAGAGTAAA TGTCATAAGG ATGCCAGTCT GTGTAGAGAT TGATGTGTTA
21481 CTAGCAGATT CATGAAATAA AGGCTGAGGA TGTAGTCCCC AAGTCACTTC TGAGTGGAAG
21541 AATTTCTCCT TTGTCCTGGA CTCAAATATT TTAGGATAAA GGAAAAAAGA AGATATTTAT
21601 AGAAGGGACT TGTTTTCAAG TACTTGACAA AATTTCACCA TTAAAGAGAA ATTTGTGGGA
21661 GTTCCCATCG TGGCTCAGTG GAAACAAATC CAACTAGGAA CCATGAGGTT GTGGGTTTGA
21721 TCCCTGGCCT CACTCAGTGG GTTAAGGATC CGGTGTTGCC GTGAGCTGTG GTGTAGGTTG
21781 CAGACACGGT TCTGATCCTG CGTTGCTGTG GCTGTGGCTG TGGTGTAGGC CAGCAGCAAA
21841 CAGCTCTGAT TAGACCCCTA GCCTGGAAAC CTCCATATGC CACAGGTGCA GCCCTAAAAA
21901 GACAAAAAAA GAGAAAAGAC AAAAAAAAAA AAAAAAAAAA AAAAAAAAAA AAAAAAAAAN
21961 NNNNNNNNNN NNNNNNNNNN NNNNNNNNNN NNNNNNNNNN NNNNNNNNNN NNNNNNNNNN
22021 NNNNNNNNNN NNNNNNNNNN NNNNNNNNNN NNNNNNNNNA GAACCACCAG AGGTATTTAT
22081 TTGTTTTTGC CTTTTTTCAC TGACTGTTTT TTGTTTGTTT GTTTGAGACT GATCTAGAAG
22141 ACTAGAGATT ACAAGAAATA TGGATTTGGC TCACTCTAAG AAACTGCTTT CATTCCAAGG

22201 TTTGGGTCTA TCCAAAAGTG GAATAGAATC ATATGAATAC TAGTTTATGA GTATTTAGTG

22261 AGAGGAATTT CAAGCTCAAA TAATGATTCA GCAAGATTAA ATTAAGGAGG GAATTTTCCT

22321 TGTGGCTGAG TGGGTTAAGG ACCCAATGTT GTCTCTGTGA GGATGTAGGT TCCATCCTGG

22381 GCTTTGCTCA TTAGGTTAAG GATCTGGCAT TGCTGCAGCT CAGACCCAGT GCTGCCCTGG

22441 TTGTGGCTTA GGCCAAAGCT GCAGCTCCAA TTCAATCTCT GGCCTGGGAA CCTCCATGTG

22501 CTACAAGGTG CGGCCTTAAA AGGAAAAAAA AAAAAATTAA ATCAAGGACT CAAGAGTCTT

22561 TCATTATTTG TGTTGTGGAA GCTATATTTG TTTTAAAGTC TTAGTTGTGT TTAGAAAGCA

22621 AGATGTTCTT CAACTCAAAT TTGGGAGGGA ACTTGTTTCA TACATTTTTA ATGGATAAGT

22681 GGCAAAATTT TCATGCTGAG GTGATCTATA GTGTTGTAAT GCAGAATATA GTCAGATCTT

22741 GAACATTTTA GGAAGTTGGT GAGGGCCAAT TGTGTATCTG TGCCATGCTG ATAAGAATGT

22801 CAAGGGATCA CAAGAATTCG TGTTATTTGA CAGCAGTCAT CTTTAAAAGG CATTTGAGAA

22861 AGTCCAATTT CAAATGCATT TCCTTTCTTT AAAAGATAAA TTGAAGAAAA TAAGTCTTTA

22921 TTTCCCAAGT AAATTGAATT GCCTCTCAGT CTGTTAAAAG AAACTCTTAC CTTGATGATT

22981 GCGCTCTTAA CCTGGCAAAG ATTGTCTTTA AAATCTGAGC TCCATGTCTT CTGCTTTATT

23041 TCTGGTGTGC CTTTGACTCC A***GATTACAGT*** ***AAATGGAGGA*** ***C***TGAGTATAG GGCTAAAAAG
GAT|T cut. ***sgSL27***

23101 TAGAGAGAAT GGATGCATAT TATCTGTGGT CTCCAATGTG ATGAATGAAG TAGGCAAATA

23161 CTCAAAGGAA AGAGAAAGCA TGCTCCAAGA ATTATGGGTT CCAGAAGGCA AAGTCCCAGA

23221 ATTGTCTCCA GGGAAGGACA GGGAGGTCTA ***GAATCGGCTA*** ***AGCCCACTGT*** AGGCAGAAAA
sgSL26 ***cut. C|TGT***

23281 ACCAAGAGGC ATGAATGGCT TCCCTTTCTC ACTTTTCACT CTCTGGCTTA CTCCTATCAT

23341 GAAGGAAAAT ATTGGAATCA TATTCTCCCT ***CACCGAAATG*** ***CTATTTTTCA*** G**CCCACAGGA**
*C**AC|C cut.*** ***sgSL25*** **Exon 7**

23401 **AACCCAGGCT GGTTGGAGGG GACATTCCCT GCTCTGGTCG TGTTGAAGTA CAACATGGAG**

23461 **ACACGTGGGG CACCGTCTGT GATTCTGACT TCTCTCTGGA GGCGGCCAGC GTGCTGTGCA**

23521 **GGGAACTACA GTGCGGCACT GTGGTTTCCC TCCTGGGGGG AGCTCACTTT GGAGAAGGAA**

23581 **GTGGACAGAT CTGGGCTGAA GAATTCCAGT GTGAGGGGCA CGAGTCCCAC CTTTCACTCT**

23641 **GCCCAGTAGC ACCCCGCCCT GACGGGACAT GTAGCCACAG CAGGGACGTC GGCGTAGTCT**

23701 **GCTCAA**GTGA GACCCAGGGA ATGTGTTCAC TTTGTT***CCCA*** ***TGCCATGAAG*** ***AGGGTA***GGGT
sgSL28 ***cut.*** ***GG|GTA***

23761 TAGGTAGTCA CAGACATCTT TTTAAAGCCC TGTCTCCTTC CAG**GATACAC** **ACAAATCCGC**
Exon 8

23821 **TTGGTGAATG GCAAGACCCC ATGTGAAGGA AGAGTGGAGC TCAACATTCT TGGGTCCTGG**

23881 **GGGTCCCTCT GCAACTCTCA CTGGGACATG GAAGATGCCC ATGTTTTATG CCAGCAGCTT**

23941 **AAATGTGGAG TTGCCCTTTC TATCCCGGGA GGAGCACCTT TTGGGAAAGG AAGTGAGCAG**

24001 **GTCTGGAGGC ACATGTTTCA CTGCACTGGG ACTGAGAAGC ACATGGGAGA TTGTTCCGTC**

24061 **ACTGCTCTGG GCGCATCACT CTGTTCTTCA GGGCAAGTGG CCTCTGTAAT CTGCTCAG**GT

24121 AAGAGAATAA GGGCAGCCAG TGATGAGCCA CTCATGACGG TGCCTTAAGA GTGGGTGTAC

24181 CTAGGAGTTC CCATTGTGGC TCAGTGGTAA CAAACTCGAC TGGTATCCAT GAGGGTATGG

24241 GTTTGATCCC TGGCCTTGCT CAATGGGTTA AGGATCCAGC ATTGCTGTGA GCTGTGGTAT

24301 AGGTTGCAGA CTCTGCTCAG GTCCCATGTT GCTGTGATTG TGGTGTAGGC TGACTGTTGC

24361 AGCTTCAATT TGACCCCTAG CCCGGGAATT TCCATAGGCC ACACGTGCAG CACTAAGGAA

24421 GGAAAAAAAA AAAAAAAAAA AAAAGAGTGG GTGTGCCTAT AGTGAAGAAC AGATGTAAAA

24481 GGGAAGTGAA AGGGATTCCC CCATTCTGAG GGATTGTGAG AAGTGTGCCA GAATATTAAC

24541 TTCATTTGAC TTGTTACAGG GAAAGTAAAC TTGACTTTCA CGGACCTCCT AGTTACCTGG

24601 TGCTTACTAT ATGTCTTCTC AGAGTACCTG ATTCATTCCC AGCCTGGTTG ACCCATCCCC

24661 CTATCTCTAT GGCTATGTTT ATCCAGAGCA CATCTATCTA ACACTCCAGC TGATCTTCCT

24721 GACACAGCTG TGGCAACCCT GGATCCTTTA ACCAACTGTG CCAGGCTGGA GATCAAACCT

24781 AAGCCTCTGC AGCAACCCAA GCTGCTGCAG TCAGATTTTT AACCCCCTGT GCCACTGTGG

24841 GTATCTCCGA TATTTTGTAT CTTCTGTGAC TGAGTGGTTT GCTGTTTGCA G**GGAACCAGA**

Exon 9

24901 **GTCAGACACT ATCCCCGTGC AATTCATCAT CCTCGGACCC ATCAAGCTCT ATTATTTCAG**

24961 **AAGAAAATGG TGTTGCCTGC ATAG**GTGAGA ATCAGTGACC AACCTATGAA AATGATCTCA

25021 ATCCTCTGAA ATGCATTTTA TTCATGTTTT ATTTCCTCTT TGCAG**GGAGT GGTCAACTTC**

Exon 10

25081 **GCCTGGTCGA TGGAGGTGGT CGTTGTGCTG GGAGAGTAGA GGTCTATCAT GAGGGCTCCT**

25141 **GGGGCACCAT CTGTGATGAC AGCTGGGACC TGAATGATGC CCATGTGGTG TGCAAACAGC**

25201 **TGAGCTGTGG ATGGGCCATT AATGCCACTG GTTCTGCTCA TTTTGGGGAA GGAACAGGGC**

25261 **CCATTTGGCT GGATGAGATA AACTGTAATG GAAAAGAATC TCATATTTGG CAATGCCACT**

25321 **CACATGGTTG GGGGCGGCAC AATTGCAGGC ATAAGGAGGA TGCAGGAGTC ATCTGCTCGG**

25381 GTAAGTTCTG CACATCACTT CGGGTTACAA TGATTTAAGA AACAACTAAG GTGGGGCAAA

25441 GGGTAGTGAG GCATATCCAT CAGAGCAAAT TCCTTGAAAT ACGGACTCAG AGGAAACCAT

25501 TGTGAGATTG AGGTTCCCAG AGGTGTGGAT TTAATGAATT AGTGTTACCT CATGTACAAG

25561 GTAGTATACT ACCAGAAAGA TAAAAATTCA GAAGCGAGTT TGCAGCAAAA CTCATAGGGA

25621 GAACTTCTTT TATAAATAAT ATGAAGCTGG ATATTTAGTG CACCACCTGA TGACCACTTT

25681 ATTAATAAAT AAAGAGTTCC TGTTGTGGCG CAGCGGAAAT GAATCCGACA AATAATCATG

25741 AGTTTGCGGG TTTGATCCCT GACCTCGCTC AGTGGGTTGG GGATCTGGTG TTGCCATGAG

25801 CTGTGGTGTA GGTCGCAGAT GCTGCTTGGA TCCCGCTTTG CTGTGGCTGT GGTATAGTCT

25861 TGTGGCTACA GCTCCGATTT GACCGCTAGC CTGGGAACCT CCATATGCTG CGGGGGTGGC

25921 CCTCAAAAGC AAAATAAATA AATAAGTAAA TAAATAAGTA GTTTAAAAAG GACAAGAAGA

25981 AATATATTTG GTATTATATT CTACAGAGAC AAAGATAATC ACCATGCCCG ATTGATTTTT

26041 CAAGGCATAT AAATGAGACG TCATGGGAGC AAAAATGGTC ATAATACAAT GCCCTTGTTT

26101 TGTGTACATG GTAAGATTTT AGAAAGCATT GTGAAGTAGA AAAGTGTACT CAGTTATAAT

26161 ATATTGGAGA AAACAGTACT ATGAGAAGTA AAAAAATCTA CATGCCGGAA TTTATTTTTT

26221 TAATGTCTCT TTAGAGTCGC ACATGCGGCA TGTGGAGGTT CCCAGGCTAG GGGTCGAATC

26281 AGAGCTATAG CCACTGGCTT ATGGCACAGC CACAACAACG CTAGATCTGA GCCACATCAG

26341 AGACCTATAC TATAGCTCAT GGCAATGCCA GATCCTTAAC CTACTGAGCC AAGCCATGGG

26401 TCAAATCCAG GTCCTCATGG ATCCTAGGCA AATTCATTTC TGCTGAGCCA CGAAGGGAAC

26461 TCCTCAGAAG TGATTTTGAT GTTACTTTCT TTTCATGACA AATCTGGTAA AGTACATACA

26521 CATAGAAACT GAAGTGTCAG AAAGGGAAAT ATTTCATTTT AAGGTAATGT ATACAAAACA

26581 GTGGTTTTAC CATCTGAGTA TCTTGCTAAA TTTTAACTAT CAAGGACAAT TGCCAAAAAA

26641 AAAAAAAAAA GAGAGAGAGA GAGAACAGAA TAGGGTTATG AAGCTAAAAT CACAGGGTTA

26701 TGAAGCTAAA ATCACAGTAA TTTAGGGAGA AAAAAATCCA AAGCATGTAA TTGATAAAAG

26761 GCTCTGAGCC TTTGTTTGAG ATTTAGAATT CAACTTGGAA ATACCGGTGG TATTTTAAAG

26821 CAGTCCATAA GTATAAAATC CAAGGCTAAA AAGCCAGAAG GTATTTGTAG AACAAATATA

26881 TTTTAATAAG CTCTACCAAG TCATCCAGAA GCTACTAAAG AATTACTGGT CACTGACATA

26941 GTGTACCTGT TTTCAAGGCC ATTCTTACAT CAGAATAAAG GGAGAGCACC CTCTGAATCT

27001 TCAGAAAAGA TGTGAAAGTG CTAATTCTCT ATTTCATCCC AG**AGTTCATG** **TCTCTCAGAC**

Exon 11

27061 **TGATCAGTGA AAACAGCAGA GAGACCTGTG CAGGGCGCCT GGAAGTTTTT TACAACGGAG**

27121 **CTTGGGGCAG CGTTGGCAGG AATAGCATGT CTCCAGCCAC AGTGGGGGTG GTATGCAGGC**

27181 **AGCTGGGCTG TGCAGACAGA GGGGACATCA GCCCTGCATC TTCAGACAAG ACAGTGTCCA**

27241 **GGCACATGTG GGTGGACAAT GTTCAGTGTC CTAAAGGACC TGACACCCTA TGGCAGTGCC**

27301 **CATCATCTCC ATGGAAGAAG AGACTGGCCA GCCCCTCAGA GGAGACATGG ATCACATGTG**

27361 **CCA**GTGAGTA TCCATTCTTT AGCGCCACTG TTATCTTCTG ATCTACCTAA GCAGAAGTGT

27421 TATAACCTTT AGATAATCCC TATTCTACCT GGATGATGAG ATTCATTCTC TTTAATTTGG

27481 TGTGCAGGTA TTCAGGATCA GTGATCATTT TCCCAAAGAC CATCATGCTC TGATGGTCTT

27541 CTCAAAAGTT CTAATCAGTT GCTTCCTCCG TGAACAGTTG AGGAGCAGAG AATATGTAAT

27601 TCAGAATTTG ACTATTGAAT CATCCCATTT TTCTTTCACA TAGTCTTTTG TTGCACTGAG

27661 TATAAGGAGA GAAGCAGTCA GAAAGATCAA TCCTGAATTA TTTCTCCATT CTACATCTGT

27721 TTTAAATTTC AAAAAAAATT GTTATAGGTG ATTTACAATG TCTGTCAATT TCTGCTCTAC

27781 AGCAAAGTGA CCCAGTTATT TACATATACA TTCTTTTTCT CATATTTTTA AACCGGGAGA

27841 TTTCTATCCA CCTGGCAGTT TGAGGGAATT TAACATTATG CATTTATGTT AACTTTATTC

27901 ACCTGATGTT TTCTAAGTCA TACTGAGATT CTTATGTCCA GGATGGAATA CACCTGGTTT

27961 GCTGGAAAGA CATGTGCTTT CATAAAGATG AATTTTGGAA AAAATATAAA ATTTAAAAGT

28021 CCCATTAAAT AAGCAAAGTT TTAAGAGATT TCAAAAAAAA TTTCATCTCT CTCTTTTCCT

28081 CTTTGACCTC TTGGGCACGT TCATCTTCTC AAATATGATC TTGGTGTTTC TGACTTTTCA

28141 G**ACAAAATAA GACTTCAAGA AGGAAACACT AATTGTTCTG GACGTGTGGA GATCTGGTAC**

Exon 12

28201 **GGAGGTTCCT GGGGCACTGT GTGTGACGAC TCCTGGGACC TTGAAGATGC TCAGGTGGTG**

28261 **TGCCGACAGC TGGGCTGTGG CTCAGCTTTG GAGGCAGGAA AAGAGGCCGC ATTTGGCCAG**

28321 **GGGACTGGGC CCATATGGCT CAATGAAGTG AAGTGCAAGG GGAATGAAAC CTCCTTGTGG**

28381 **GATTGTCCTG CCAGATCCTG GGGCCACAGT GACTGTGGAC ACAAGGAGGA TGCTGCTGTG**

28441 **ACGTGCTCAG** GTGAGGGCAG AGAGTCTGGA TTGAGCTTGG AAGCTCTGGC AGCAAAGAGA

28501 GGGTGGGCGG TGACCTGCAT TGGGTAAAGA TTGGAAGGTC CAGCCTAAGG ATCTGGTGGT

28561 GGGGGGAGAC ATGATGTTTC AGTCTGAAGA ATGATGAAAA CCTGTGTTGT TACGCATGGG

28621 CCTTCACCGA GGAAAGGAAC ATAACTTACA TGTATCCTCC TGCAGAGGGA GGAAGAACTA

28681 GGGGATTCTA GTTTTGTGTG GGAAGGAGCA GTTTACTTGG TTCAGGAGGC ACTAAAGGCT

28741 CAGATAGGAA ACAGAGATCT GTTCCATTCT TACTCCCAGA ACTGATTCTC TTCTCTTTTC

28801 TCCTACAG**AA ATTGCAAAGA GCCGAGAATC CCTACATGCC ACAG**GTATAT AAAAAAGTTT

Exon 13

28861 AAGAACATGG GACCCATTGT CTGCATTTTG TGGAATCCCT CTTATTAAGA CATTCTGGGT

28921 CAGAAGTTCT GAGGATTTGA CATTTACTTC AGCTATCTGT TATCTTACCC AAGAGAGGGA

28981 TGGTAACTAG GAACCCAGGT CTTTTAGCTA AGACATTATC ACCTCTTGTG ATGTTTACTT

29041 GTTCTCAG**GT CGCTCATCTT TTGTTGCACT TGCAATCTTT GGGGTCATTC TGTTGGCCTG**

Exon 14

29101 **TCTCATCGCA TTCCTCATTT GGACTCAGAA GCGAAGACAG AGGCAGCGGC TCTCAG**GTCT

29161 GAACAAAATT ACGGTCTCTC TAATGTTTCT ATGGGATAAG AAGCCTCTCT GGATAATAAA

29221 ACAAAAAAAT TACATTCAAG TATCAGTTGG CCAGAAAGAG GGAACCTAGA AGAGGTTTAA

29281 GCAGTTTCTC CGAAACAGGG AACAAGAATT CAGAGAAGAA AAGGCACATT GGCTGTACTG

29341 ATGATACCTG CACTCGCTAT GTATGTTTAA TGGGGGACAG TAGAGAATTG ATAGTTTAGA

29401 AGGAGTATGC TTATATGGTT CTGGATGAAT CCTGTATCCC CCCAAACATT TATTTTCTCT

29461 TACTATATAC TTATTACTAA TTTAACTCTT CTGTCAAGCC GTGTGCTAGG TTCTGAAGAT

29521 GGTTCAGACT TGGATACTCA AGTGCTTTTG TTTTCATGGA ATTTCCAGTT TAGTGGAAGA

29581 GATAAATATG TAAACAAATA AATTGCAATG TTTTATTATA CATTCGTGTG AATAAGGAAC

29641 AAAGGAGGCA CAGAGAATAA AGTAATTACT GAAAGGGGAA GGGGAGTATC AGAGACTTCT

29701 AAGTTTGGAG GCAGATTTTG AAGACAGAAA TCAAAGTACT GGGTAAGATG CATTTCAGGA

29761 AAGAAGAAAA ATATGTACAC GTGTAGAGAA GCTTAAAAGA GGGCACATTT GTTGTTTTGG

29821 AGGGGAGTAC AAGTTGAGTT AAAGAGAGAA GTTTCTGTTA AGGCTGAAGA ATAGGGAAGA

29881 TACACGTAGC GATGCTCTGT GTTGCATGAT AAGAAGAGTC GGAGTTATTA AAGAGTATGA

29941 GATAGGGGAG TGAGATAGGC AGGCAGGTCC TTAGAAAGTT CTGTTTGGAA ATGGGATGTC

30001 GGAGGGGTTG AAAGAGAACC ATATATTGAC AAGGAGAGCA TTTTGAAGTA GTTGTGATGA

30061 AAGATAAAAT GGACTTTATA GTGAGAATGG CTGGGAAAGG ATAGATTTTA TACAAATCTC

30121 CAATGAATTA CAGAAGAATG CTACCTGTCT TTGGGGAAGA AACAGGGTTA TCCGATGGCA

30181 TCCTGTTGCG TTTGAGTTCG TGACATCATG AGGGAAAGGC TTGGCAGCGT TTACTCGGTA

30241 CTGTGTGGTA ACTTATATGG AAAAAAATAT GAGAAGGAAT GAGTGTGTGT ATAACTAATT

30301 TACTTAGCTG TATGCCTGAA ATTAATACAA TTTTATAAGT CAACTCTACT CCAATAAAAC

30361 AAACAAATAA ATAAATAATT TTAACTACCT GAACAAAAAA AAAAGAATGG ACTGGAGACA

30421 AGTCAAAAGT ATGGATGATG ACTACGTTAT GCTTGCACTG CTGGGGAAAA GCACACATAG

30481 GGAGGGAACG TTTTATTATG ACCCAGTCCC TAACCTATGA CCTCTGTTAT CAG**TTTTCTC**

Exon 15

30541 **AGGAGGAGAG AATTCTGTCC ATCAAATTCA ATACCGGGAG ATGAATTCTT GCCTGAAAGC**

30601 **AGATGAAACG GATATGCTAA ATCCCTCAG**G TCCGTGGGTT CTTTGAGGGC CTGTAGCCCT

30661 GGGGTTCAGA TCAGCAGCTG CAGTTGAGGT TGAGGCATGC TACTTTGCAC AGCAGTAGAA

30721 AGAAATCTCA ACTGTAATAG GAAGCTTGGG ATGCATATGA GGAAGAAAGG CAAGAATGAA

30781 CCACAAATTA TTCTTAGGGA AGATAAAAAT TGCAGTCATG GGGAGACCTC TGGCTGAGAG

30841 GGCCGTGATT ATTTCTGACA GAGGGATTAT GGAGTAGAAT ATGATGGCTT GGACCTTTTT

30901 TCACTAAAAC AAGTCAGTCT TCTCAAAGGT AGTTTAGCTT TTCATATATC TTTCTCAGTT

30961 TCTTCCATTC CCATTTCCTG CCATTTTCCT TTCTCTAACT TTTATTTATT ATATTTTTTC

31021 CTAAAAGTTT AAATTTTCTA TATCTTTATC CCTTCAGAAG CCATCCCTAG TCACAGGACT

31081 AGTTTTATTT CCCATTATGT AATGCTTCTT TCTCTGTCTG TTGACTTCTA TTTAGAACCA

31141 GTGCACTAAA TCTGCCTCTA GGAACATACC TCTGCTAGGT TGCAAGAAAT ATCCCATTCC

31201 CCACTCACTC TGTGAAGACT CAATGCTTCT CAATATTCCT TACCTCCTGA GAGGGACTTG

31261 CCTCACTTCT TTAATCCAAG GGACTCGATT TTTGCCAAAA CTAAGTCAGG AAAACCTACA

31321 TAAGACATAG GAAAGACTTG CTGTGCTTCT TAAACCCCAC TGTTTGTTTT CCTAATTGTG

31381 AACAGTATTT TTAAAGTTCA AAGAGCTTCT AAGGCACTTG AGGGGAGATC TGATTTATTT

31441 CCCAGTAATT ATTTTATTCC TTTCAGAAAA TTCCAATGAA TAAGATGGTT TTAATGATGT

31501 GGGACTAATT TTTGTGTCTA AATCTCTTCC TATTTCTGGA TGAAAAAAAG **GAGACCACTC**

Exon 16

31561 **TGAAGTACAA TGA**AAAGGAA AATGGGAATT ATAACCTGGT GAGGTGAGTA AAAAGAATTT

31621 ATTCATCATT GCTGAAAACA GGTACATTCC TTTTGAAAGT TGGGAACTCC TCTGGTATTA

31681 GAAAAAAAAA AAAGAACGTA TATACACATA TATTTCCATG TCTATGTTTA TGTTTGTAAA

31741 TCCATATTCA GAATATGCAA CAACTTTTTA TAACTATGAC TTCAGTCCAT CTTTTAGTTA

31801 CATATATATT CTAAACAACA ACTATTGCTA AGAGAAGCTG GGTAAGTAAA TGTGAATAAA

31861 TCTTCTAAAG ATATTACAGG AAGTTCCTGC TGCGGCTCAG TGGGTTAAGG ACTTGATGTC

31921 TTTGTGAAGA TGAGGGCTCG AGCCCTGGCC TCACTCAGTG AGTTAAGGAT CTAGCATTGC

31981 TGTAAGCTGC AGCGTAGGTT GCAGATGGGG CTCAGATCCA GTGTTGCTGT GGCTGTGGCC

32041 TCAGTTGCAG CTCTGATTCA ACCCTTAGGC GAGGAACTTC CATATGCAGC AAATGTGGCC

32101 ATTAAAAAAA AACAAAAAAC ATTATAGGAG TCATTTCATA AAAGAGATAA GACGTTTCTA

32161 TAGTTATATA GTGCATACTC TGGTAAAGAT AGTATAGGAT ACTATAGGAA TATAGAAAGC

32221 TTGCCTATGA AAATTTGGGA AGATTGTGGA AAAGACATCT CAAAATATGG CATAGAAAAG

32281 AATCATATCT TTGAGGAACA GTAAGTTTTT CATTCAAAAC CGTGTATTGA ACATACTTAT

32341 GGTGACAAAT GGTGTCTTGA GTACTAAAAA TTCAGTGATA AAAGATGCTC TTGACAAAGA

32401 CATGGCTGTT GAATAGAAGG TCTCACTGTC AATGTGTGGG AATTATGGAC AGCCTATGTG

32461 GACACAGGGA ATAGATGAGA CTCTAGGCTG GAAGGCTGCA TTGAGCCCAG TAATGAATGG

32521 TCCTGTCTGA TATATTTCAT GCTCATATTT TATTTTAGGG ACTATTGGGG AGGTGGTGGG

32581 CTTTGGAAGA TTAAGCTGAG GCAAGACACA ATCAGATTGC CTTTTATAAT TTACTTTCAG

32641 GAGGAAAATC TAACTAAAGA AAAAAAGTGA ATAAGGCAAG AAACATAAGT TATACATCAA

32701 AAAGAAAAGG TAGTGGAGTT CCTGTTGTGG CTCAGTGGTT AATGAACCCT GCTAGGAACC

32761 ATGAGGTTGT GGGTTCGATC CCTGGCCTTG CTCAGTGGGT TAAGGATCCA GCGATGCCAT

32821 GAGTTGTGGT GTAGGTCGCA GACCGTGGCT TGGGTCCCGC ATTGCTGTGG CTATGGTGTT

32881 GGCTGGCAGC TGCAGACAGC TCTGATTA
